# Biodegradable Biopolymeric Nanoparticles for Biomedical Applications-Challenges and Future Outlook

**DOI:** 10.3390/ma16062364

**Published:** 2023-03-15

**Authors:** Radhakrishnan Sreena, Arputharaj Joseph Nathanael

**Affiliations:** 1 Centre for Biomaterials, Cellular and Molecular Theranostics (CBCMT), Vellore Institute of Technology (VIT), Vellore 632014, Tamil Nadu, India; 2 School of Biosciences & Technology (SBST), Vellore Institute of Technology (VIT), Vellore 632014, Tamil Nadu, India

**Keywords:** biopolymers, biopolymeric nanoparticles, biomedical, tissue engineering, drug delivery

## Abstract

Biopolymers are polymers obtained from either renewable or non-renewable sources and are the most suitable candidate for tailor-made nanoparticles owing to their biocompatibility, biodegradability, low toxicity and immunogenicity. Biopolymeric nanoparticles (BPn) can be classified as natural (polysaccharide and protein based) and synthetic on the basis of their origin. They have been gaining wide interest in biomedical applications such as tissue engineering, drug delivery, imaging and cancer therapy. BPn can be synthesized by various fabrication strategies such as emulsification, ionic gelation, nanoprecipitation, electrospray drying and so on. The main aim of the review is to understand the use of nanoparticles obtained from biodegradable biopolymers for various biomedical applications. There are very few reviews highlighting biopolymeric nanoparticles employed for medical applications; this review is an attempt to explore the possibilities of using these materials for various biomedical applications. This review highlights protein based (albumin, gelatin, collagen, silk fibroin); polysaccharide based (chitosan, starch, alginate, dextran) and synthetic (Poly lactic acid, Poly vinyl alcohol, Poly caprolactone) BPn that has recently been used in many applications. The fabrication strategies of different BPn are also being highlighted. The future perspective and the challenges faced in employing biopolymeric nanoparticles are also reviewed.

## 1. Introduction

Nanotechnology is the study that involves designing or fabricating materials and devices with at least one dimension of one billionth of a meter [1]. Multiple researchers have proved the advantages of the nano-dimension over the micrometer scale owing to the enhanced individual molecule interaction compared to the bulk [2]. Nanoparticles are zero-dimensional nanomaterials (0D) with a size range from 10 to 1000 nm. They are employed in many biomedical applications such as drug delivery [3], tissue engineering [4], biosensors [5], gene delivery [6], cell imaging and labeling [7,8] because of their enhanced surface-to-volume ratio and magnetic properties [9]. Nanoparticles have created an important role in the advancement of therapeutic applications since they exist in the same size range as that of proteins, and their small size and large surface help in the exposure of surface functional groups that can be tailored according to the requirement [10]. Nanoparticles obtained from biological sources are highly preferred because of their improved quality and stability compared to metal-based nanoparticles, where most are toxic to the human system [11]. Thus, nanoparticles can be obtained from biopolymers as a solution to the disadvantages posed by the counter-sources [2]. 

**Figure 1:**
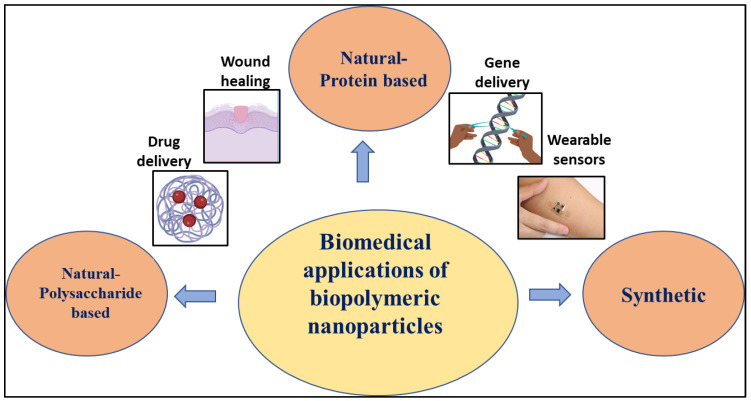
Schematic representation of the biomedical applications of biopolymeric nanoparticles.

Biopolymers are the polymers obtained from living organisms such as plants, animals or microbes; they also include synthetic polymers obtained from renewable feedstock, bio-based monomers and also fossil fuels. Biopolymers can be classified into polysaccharides, polypeptides and polynucleotides based on the monomeric unit of the polymer, and are available in abundance and used extensively in the biomedical field, such as for wound healing drug/gene delivery, tissue engineering and cell imaging [12]. BPn appears to offer a solution to ameliorate the environmental effects and issues in biocompatibility and biodegradability caused by synthetic materials. The most important parameters that have a crucial impact on the fabrication of BPn are the surface charge, size, stability, compatibility with the cells and degradation [13]. Albumin was the first fabricated BPn [2]. Though polymeric nanoparticles have an issue of scaling-up and their capacity of drug loading is also comparatively low, researchers have widely employed them and tried ways to combat the disadvantages [10]. They have favorable properties such as biocompatibility, good anti-oxidant and anti-bacterial properties, and tailorable surface features [11]. BPn acquired from proteins and polysaccharides are superior when compared to synthetic materials, as the former can be easily metabolized naturally by the enzymes present in the digestive system, whereas the latter accumulates and leads to the formation of toxic by-products. Protein-based BPn can be surface modified, which can facilitate site-directed drug targeting [14,15]. One of the main limitations in employing biopolymeric nanoparticles from proteins or nucleic acids is that they are hydrophilic, whereas the polymers are mostly hydrophobic in nature and thus cause difficulties in drug encapsulation and degradation. Therefore, the preparation of biopolymeric nanoparticles is extremely critical [2]. Biodegradation of natural polymers occurs through biological processes, including enzymes such as collagenase in vivo and also via non-biological processes such as hydrolysis. It has been reported that the majority of natural polymers degrade with the help of enzymes. Polysaccharide-based biopolymers are degraded enzymatically within the human system with the help of enzymes such as lysozymes and amylases. Biodegradable synthetic biopolymeric nanoparticles degrade by hydrolysis of esters or urea linkages. It is also reported that polymers with polar groups degrade faster when compared to those with non-polar groups [16]. Table 1 shows a summary of the advantages and disadvantages of different sources of biopolymeric nanoparticles. Surface modification of the BPn is carried out to fine-tune the properties of the fabricated nanoparticles employed for biomedical applications. Some of the strategies employed include physical immobilization; modifications using chemicals such as grafting with amino, acrylate or acetyl group; and grafting induced by radiation such as ultrasonic waves. This type of modification enables improvement of the stability and the activity of the BPn and also aids in preventing aggregation, protecting them from any alteration [17]. BPn can be fabricated by employing different methods such as coacervation, desolvation and electro-spray techniques without employing the use of harsh organic solvents [13]. Figure 1 shows the schematic representation of biopolymeric nanoparticles employed for various applications. This review highlights the various biopolymeric nanoparticles employed for biomedical applications such as tissue engineering, drug delivery and images, and the various fabrication strategies are also discussed. The current status and the challenges in employing them are also highlighted.

## 2. Protein Based Biopolymeric Nanoparticles

Proteins are basically made of amino acids linked via peptide bonds, and their structure is stabilized by means of hydrophobic interactions and hydrogen and disulphide bonds [31]. These naturally derived polymers are highly preferred because of their excellent biocompatibility and good degradation characteristics. There are no harmful by-products since the degradation process is completely natural [32]; thus, the nanoparticles derived from the protein-based biopolymers are less toxic and easy to fabricate. The surface can also be easily tuned with respect to specific drug delivery applications [33]. A few other advantages of employing protein-derived biopolymeric nanoparticles for biomedical applications are that the fabrication is comparatively easier, and it has been reported to be more stable in vivo. The size distribution can be easily controlled and the process can be scaled up [2]. The defined primary structure in protein helps in the easy attachment of various drugs that play a key role in therapeutic applications [13]. The secondary structure of the protein determines the size of the proteins and also helps to fabricate nanoparticles precisely [10]. Some examples of nanoparticle-derived protein biopolymers employed for biomedical applications include silk fibroin, albumin, gelatin and collagen, which will be discussed in the following subsection.

### 2.1. Albumin

**Figure 2:**
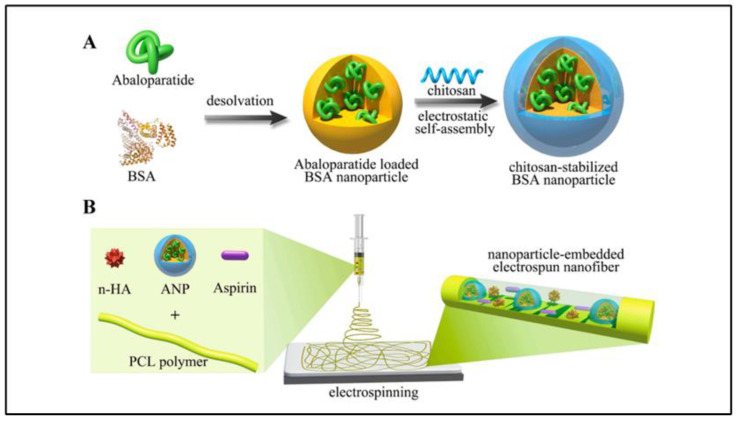
Schematic illustration of (**A**). Synthesis of chitosan stabilized BSA nanoparticle by desolvation approach; (**B**). Fabrication of PCL-based electro-spun nanofiber loaded with nano-hydroxyapatite, abaloparatide loaded BSA nanoparticle and aspirin. (Reproduced with permission from [37]; Copyright 2022, Elsevier).

Albumin belongs to the family of globular proteins and acts as a carrier protein for endogenous or exogenous compounds. It is widely employed for treating a variety of diseases—especially cancer [34]. Researchers have tested the potential of albumin in various products and clinical trials. Albumin can be easily obtained from plants, animals and human beings. Ovalbumin, bovine serum albumin (BSA) and human serum albumin (HSA) are the three commonly used albumins for biomedical applications [35]. The main advantages of employing albumin are that it has good compatibility with human cells, it does not induce toxicity, and at the same time is also biodegradable and does not cause any adverse immune reactions. Thus, albumin is a very good candidate for fabricating nanoparticles. Various proteins that are expressed in a higher range in the tumor cells, such as secreted proteins acidic and rich in cysteine (SPARC), easily and very effectively bind to albumin [18]. A study has been performed where albumin nanoparticles were employed for the simultaneous delivery of two drugs, ibrutinib (IBR) and hydroxychloroquine (HCQ), for the treatment of glioma. Drug-loaded human serum albumin (HSA) nanoparticles were prepared by ultrasonication method. HCQ, as an inhibitor, blocks autophagosome degradation. IBR has a major role in glioma treatment by suppressing the malignant tumor growth but faces disadvantages such as poor bioavailability and drug exposure in the brain cells were found to be very limited. To overcome this, drug delivery using albumin nanoparticles was facilitated. The mean size of the drug-loaded HAS nanoparticles was found to be 160.1 ± 0.7 nm. The encapsulation efficiency (%) and the drug loading capacity (%) were found to be 97.2 ± 1.8 and 3.96 ± 0.06, respectively. The biodistribution analysis showed that the presence of HAS nanoparticles resulted in an increased accumulation (5.59 times higher than free drug) of IBR drug in the tumor. The fabricated drug-loaded nanoparticles showed high cytotoxicity against C6-luc cells in CCK-8 assay and apoptosis assay. In vivo analysis in mice showed that IBR-HCQ-HAS nanoparticles stayed for a prolonged time when compared to IBR-HAS nanoparticles. Thus, these results were found to be very promising for the treatment of glioma [36]. In another reported study, abaloparatide (ANPs) was encapsulated in bovine serum albumin nanoparticles by desolvation process, stabilized in chitosan by the self-assembly process, and then made into a nanofiber scaffold for bone tissue engineering applications. Electrospinning was carried out to fabricate polymeric nanofibers from a mixture of polycaprolactone (PCL), n-hydroxyapatite (n-HAp), aspirin (ASA) and abaloparatide. The schematic illustration of the synthesis of abaloparatide encapsulated in bovine serum albumin nanoparticles and the fabrication of electro spun nanofibers loaded with two drugs is depicted in Figure 2.

**Figure 3:**
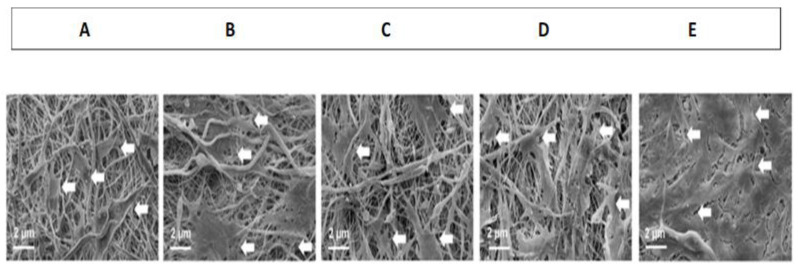
SEM images of MC3T3-E1 adhered on the nanofibrous scaffold after 2 days. (**A**) PCL/HA; (**B**) ASA/PCL/HA; (**C**) BSANps/PCL/HA; (**D**) ANPs/PCL/HA; (**E**) ANPs/ASA/PCL/HA The arrows indicate lamellipodia and cell outlines. (Reproduced with permission from [37]; Copyright 2022, Elsevier).

The size range of the chitosan–abaloparatide nanoparticles was found to be 289 ± 34 nm. The scanning electron microscopy (SEM) images of the nanofiber matrix showed that they have irregular pore structures for the diffusion of oxygen and nutrients. In vitro release studies have shown that drug release was fast in the nanofibers with two drugs when compared to the one with a single drug. The ANPs/ASA/PCL/HA nanofiber scaffold showed that the release of the drug was slow because of the hydrophilicity and degradation characteristics. Cell adhesion was studied with the help of MC3T3-E1 and the morphology was observed by SEM, as reported in Figure 3.

From Figure 3, we understand that the cells have properly spread on the nanofibrous scaffold and show fusiform morphology. Thus, the results of the dual drug-loaded nanofibers with chitosan-stabilized bovine serum albumin nanoparticles show excellent physical and chemical properties, good degradation rate, enhanced cell compatibility and osteogenic activity [37]. A study reported by Thangavel et al. used indocyanine green–paclitaxel encapsulated in human serum albumin nanoparticles that were functionalized with hyaluronic acid, as a ligand for drug delivery with image guiding capability directed to CD44 non-small cell lung cancer (NSCLC). The drug release analysis showed that paclitaxel was released more efficiently at pH 6.6 due to the acidic nature of the tumor micro-environment. Only 30% of the drug was released at pH 7.2 (blood circulation) after 46 h. The paclitaxel nanoparticles showed good anticancer activity against A549 and H299 cell lines; thus, image guided drug delivery was found to be very efficient without compromising the anticancer treatment efficiency [38]. Khella et al. studied the anti-tumor activity of MCF-7 and Caco-2 cell lines using carnosic acid encapsulated in bovine serum albumin nanoparticles. The results of the experiment showed excellent drug loading ability and the best release profile. Enhanced anti-tumor activity was found in both the cell lines; apoptosis results showed that the MCF-7 and Caco-2 cells were arrested at the G2/M phase (10.84% and 4.73%, respectively) [39]. One of the studies demonstrated the use of dexamethasone encapsulated in bovine serum albumin nanoparticles for enhanced anti-inflammatory activity in rats; a bimodal release of the drug and a significant anti-inflammatory activity was reported [40].

### 2.2. Gelatin

**Figure 4:**
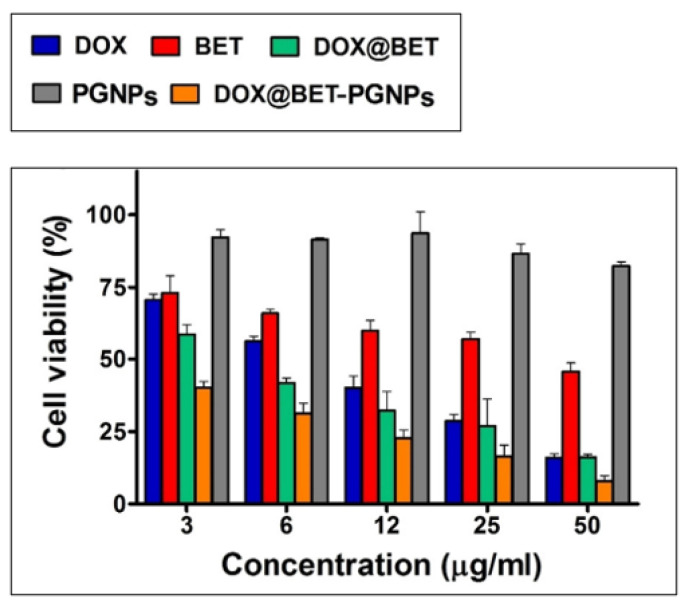
Cell toxicity results of MCF-7 cell line after treating them with individual and a combination of drugs encapsulated in gelatin nanoparticles. (DOX—doxorubicin; BET—betanin). (Reproduced with permission from [43]; Copyright 2019, Elsevier).

Gelatin is a natural biopolymer derived from animal collagen with favorable properties such as low cost, biocompatibility and biodegradability, that is derived from the hydrolysis of animal collagen [41,42]. Gelatin-based nanoparticles (GNPs) are very promising for a variety of biomedical applications such as tissue engineering and drug delivery because of their properties, such as easy availability, offering great stability and long-time storage in vivo [14]. GNPs have also been widely employed for treating brain disorders since they can cross the blood–brain barrier, and various properties such as mechanical properties, thermal and swelling behavior changes with respect to the amphoteric properties of gelatin [41]. Different cross linkers can be added to modify the physiochemical properties of gelatin nanoparticles [31]. A study reported the use of gelatin nanoparticles conjugated with polyethylene glycol for the simultaneous delivery of two drugs, doxorubicin and betanin, for cancer treatment. The particle size of the nanoparticles was found to be 162 nm. The encapsulation efficiency and the loading capacity was 82% and 20.5%, respectively. High cell cytotoxicity was observed after 48 h against the MCF-7 cancer cell line when two drugs were given rather than the individual drug, as depicted in Figure 4.

**Figure 5:**
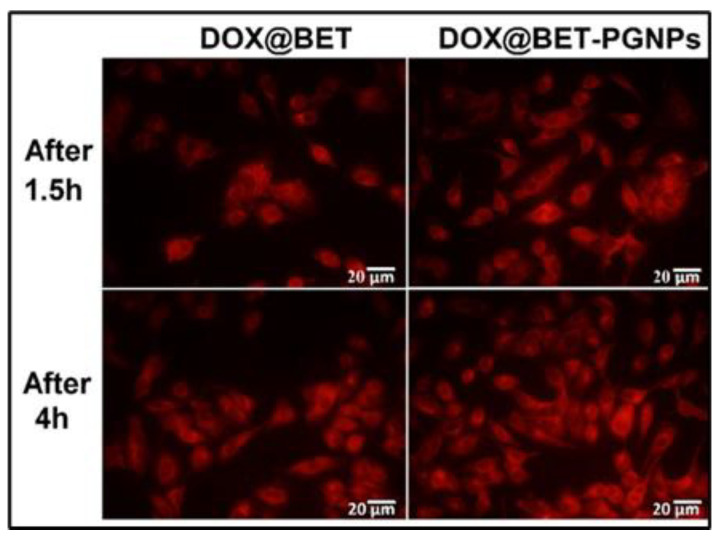
Cellular uptake studies of MCF-7 cell lines treated with the drug as well as nanoparticle encapsulated drugs. (DOX—doxorubicin; BET—betanin). (Reproduced with permission from [43]; Copyright 2019, Elsevier).

Cellular uptake results, as shown in Figure 5, revealed that high cellular uptake was witnessed with the nanoparticle-encapsulated drugs rather than the free form of the drug, proving that the nanoparticles have the ability to escape the endocytosis process.

The combination of the drugs encapsulated in gelatin nanoparticles showed excellent apoptotic activity. Thus, the multi-drug nanocarriers facilitate a new horizon to develop an enhanced treatment strategy for cancer [43]. A study reported by Yang et al. fabricated zoledronic acid (ZOL)-encapsulated gelatin nanoparticles integrated into a titanium scaffold for treating osteoporosis-based defects. The in vitro results showed enhanced osteoblast differentiation when ZOL concentration was 50 µmol L^−1^. The in vivo studies in osteoporotic rabbits showed improved bone growth and osteogenesis [44]. One study reported the use of gold nanoparticles conjugated with gelatin nanoparticles for the purpose of bioimaging as well as a drug delivery system. The size of the nanoparticles was found to be 218 nm and showed no toxicity up to 600 µg mL^−1^. The imaging of the nanoparticles in the skin tissue was carried out by using confocal laser scanning microscopy (CLSM), achieving a depth profile of 760 µm [45]. Gelatin nanoparticles were enteric coated to encapsulate 5-amino salicylic acid for oral drug delivery for the treatment of ulcerative colitis in one study. The nanoparticles’ size ranged from 225 to 250 nm and were found to be spherical in nature. The administration of nanoparticles reduced mast cell infiltration and also maintained the colon tissue architecture. A significant reduction in the inflammatory markers such as TNFα, COX-2, IL1-β and nitrate levels was observed. The encapsulated drug showed enhanced therapeutic efficiency when compared to the free drug [46].

### 2.3. Silk Fibroin

**Figure 6:**
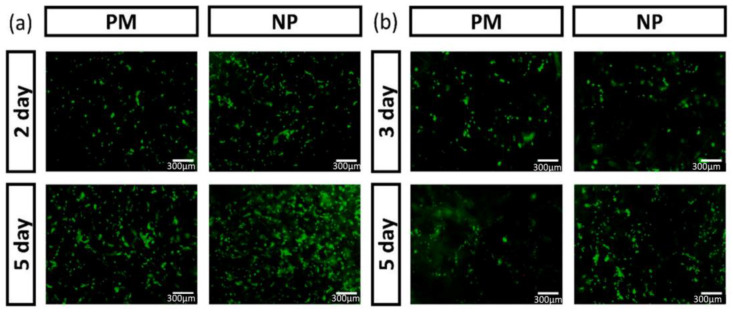
Viability and proliferation of the cells: (**a**) human umbilical vein endothelial cells (HU-VECs) (**b**) L929 cells in the scaffolds with and without nanoparticles. (Reproduced with permission from [53]; Copyright 2022, Elsevier).

Silk fibroin is a natural biopolymer obtained from the cocoons of Bombyx mori made of 5507 amino acid residues. The most important features that make silk fibroin an outstanding material for biomedical applications are its good biocompatibility with humans, very high mechanical strength and favorable biodegradable properties. It also helps to promote cell adhesion and proliferation. They can be used in various forms, such as films, hydrogels, fibers, spheres, mats, sponges and scaffolds, and are widely used in many applications such as wound healing; cancer therapy; drug delivery; and bone, skin and cartilage regeneration [47,48,49]. The controlled degradation rate and excellent biocompatibility of silk fibroin make it an excellent candidate for making nanoparticles [50]. In the nanoscale, silk fibroin shows improved physiochemical, mechanical and biological properties [51]. In one reported study, curcumin was encapsulated in silk fibroin nanoparticles for treating cancer; the therapeutic efficiency of the drug was enhanced by loading in a nanocarrier. The size of the nanoparticles ranged from 155 to 170 nm. The in vitro cytotoxicity assays revealed that the nanoparticles greatly reduced the viability of carcinogenic cells, and high cytotoxicity was seen more in neuroblastoma cells than hepatocarcinoma cells. The drug curcumin was found to be fluorescent when it was loaded into silk fibroin nanoparticles and not in the free state. The drug-loaded nanoparticles showed excellent anti-tumor and anti-oxidant activity [52]. Shen et al. developed a scaffold made of sodium alginate and silk fibroin loaded with silk fibroin nanoparticles for improving hemostasis and cell adhesion. The nanoparticles were obtained by the self-assembly process. The addition of nanoparticles to the scaffold system improved the compression strength, and reduced the degrading rate. The nanoparticles were found to be spherical and uniform in size. The cell adhesion and cell proliferation of L929 cells and HUVECs were studied by using a Live/Dead assay kit (Figure 6). It was found that at the end of 5 days, the cells showed proper adhesion, spreading, migration and proliferation. A greater number of cells were grown on the composite scaffold with silk fibroin nanoparticles (NP) when compared to the one without them (PM).

Another study reported the use of simvastatin loaded into silk fibroin nanoparticles for the purpose of bone regeneration. The nanoparticles were found in the size range of 174 ± 4 nm and were spherical in morphology. A sustained drug release profile was seen for about 35 days. The in vitro cell studies revealed that the nanoparticles improved cell adhesion and proliferation, and also showed good alkaline phosphatase activity [54]. Rahmani et al. investigated the use of silk fibroin nanoparticles for the delivery of 5-fluoro uracil for the treatment of cancer. The size of the nanoparticles was found to be 286.7 nm and the loading efficiency was 52.32%. High loading efficiency and slow release of the drug were observed [55]. Doxorubicin and PX478 were co-loaded into silk fibroin nanoparticles that were functionalized with folic acid for the purpose of treating multi-drug-resistant tumors. The cellular uptake was increased and this nanoparticle combination significantly downregulated multiple genes to overcome multi-drug resistance. The lysosomal escape was achieved quickly, and doxorubicin could quickly enter the cells and kill the drug-resistant cells [56].

### 2.4. Collagen

Collagen is a structural biopolymer that is found abundant in the human body. It is the major part of the extracellular matrix and is found in tendons, ligaments, cartilage and skin [57,58]. Collagen has been widely employed in biomedical applications due to its properties such as biocompatibility, biodegradability, favorable gelling and surface behavior [59,60]. Nano collagen has an outstanding potential when compared to three-dimensional collagens in helping to withstand heavy loads with minimum tension due to the high surface-to-volume ratio [61]. The nanocollagen has notable properties, such as high contact area, reduced toxicity, easily sterilizable, increased retention of cells, and decreased effects of toxicity from the by-products as a result of degradation. They can be found in various forms, such as sheets, films, sponges, fibers, pellets, disks and nanoparticles [62,63]. One study reported the use of collagen nanoparticles from marine sponges fabricated by the process of alkaline hydrolysis. Estradiol–hemihydrate was loaded into the nanoparticle and the drug loading was found to be 13.1%. Prolonged drug release and improved drug absorption by the cells were observed. Thus, the presence of collagen nanoparticles facilitates exciting ways of drug delivery [64]. Appropriate cross-linking strategies have to be chosen to tailor the properties of collagen according to the intended application. The stability and degradation characteristics can be altered when the surface features are altered [31].

### 2.5. Elastin

Elastin is a natural biopolymer found in elastic fibers, especially in the extracellular matrix of skin, lungs, heart and blood vessels [65]. One of the main properties of elastin is that it can retain its original shape and insolubility even after stretching [66]. They are not always biocompatible and are very much difficult to alter. Thus, soluble elastin-like peptides are fabricated for a wide variety of biomedical applications [67]. Elastin nanoparticles have been employed as a nanocarrier for delivery drugs and genes and have proven to be very effective. The ability of elastin nanoparticles to self-assemble and respond to varying temperatures has allowed them to be employed for various therapeutic applications. The properties of the elastin nanoparticles can be tailored according to the intended application [14,67,68]. One study reported the use of elastin nanoparticles for the delivery of bone morphogenic proteins (BMPs). Poly (L-valine-L-proline-L-alanine-L-valine-L-glycine) pentapeptide is an elastin-like polymer where the central glycine molecule is replaced by alanine. A total of 94% of the BMP was successfully encapsulated into elastin-like polymer nanoparticles. The in vitro assays revealed that they are non-toxic and compatible with C2C12 cells [69]. Kim et al. reported a study where α-elastin nanoparticles were fabricated for protein delivery applications. The nanoparticles were grafted with polyethylene glycol to improve the colloidal stability; they were in the size range from 330 ± 33 nm. A sustained release of encapsulated insulin and bovine serum albumin (BSA) was observed for 72 h. The thermoresponsive nature enables the fabricated nanoparticles to be employed for a wide variety of drug delivery and tissue engineering applications [70]. The summary of protein based biopolymeric nanoparticles is given in Table 2.

## 3. Polysaccharide Based Polymeric Nanoparticles

Polysaccharides are long carbohydrate molecules made of monosaccharide units that keep repeating and are linked by glycosidic bonds. Some examples of polysaccharides include chitosan, alginate, dextran, starch, heparin and hyaluronic acid. These naturally derived biopolymers form the main constituent of the extracellular matrix. The main advantages of polysaccharides are that they are highly stable, compatible with human cells and have favorable degradable properties. Carbohydrate-based nanoparticles, along with immobilization techniques, help in improving biocompatibility. Due to their small size and high surface-to-volume ratio, nanoparticles have wide applications, such as delivering drugs, proteins and nucleic acids. Polysaccharide-based nanoparticles can be fabricated by various methods and the properties can be tailored by modifying the structure according to the intended application [2,10,13,15]

### 3.1. Chitosan

**Figure 7:**
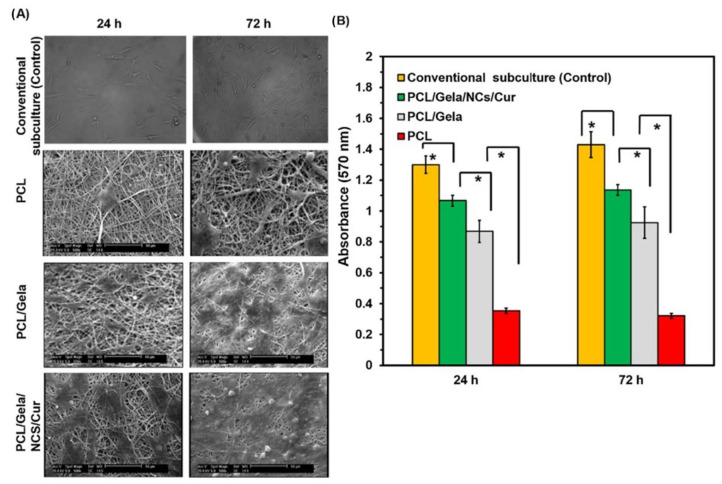
(**A**) Scanning electron microscopy images of human endometrial stem cells attached in PCL, PCL/gelatin and PCL/gelatin/chitosan nanoparticles/curcumin-loaded fibrous mats for 24 h and 72 h; (**B**) Results of cellular growth obtained through MTT assay. (Vertical bars: standard deviations; * *p*-value < 0.05) (Reproduced with permission from [87]; Copyright 2020, Elsevier).

One of the most important cationic biopolymers employed for various biomedical applications is chitosan. This hetero polymer is made of N-acetyl-D-glucosamine, which is an acetylated unit, and D-glucosamine, which is a deacetylated unit linked by β-1,4 linkages. It is a hydrophilic biopolymer with the ability to open tight junctions of the cell membranes that are degraded by the presence of enzymes such as lysozymes, proteases and lipases [10,79]. The positive charge of the chitosan nanoparticles is due to the presence of amine groups that has the ability to adhere to the negatively charged mucosal membrane and aid in the release of the encapsulated drugs in a sustained manner. A complex formation is induced by the electrostatic interactions along with hydrogen bonding and hydrophobic interactions, and thus, the mucoadhesive property of the chitosan nanoparticles are highly exploited for oral drug delivery applications. The nanoparticles also have cell compatibility in both in vitro and in vivo models [80]. The bioavailability and stability issues are overcome with the surface modification of the chitosan nanoparticles. The chitosan nanoparticles show improved bioavailability, increased specificity and reduced toxicity, and the properties vary with size. Due to all these properties, they are employed in applications such as nanomedicine, biomedical and pharmaceutical industries [81]. They can be fabricated by a variety of methods such as emulsification, precipitation, ionic or covalent cross-linking, solvent diffusion method and solvent evaporation [82,83]. Dev et al. fabricated chitosan nanoparticles along with Poly lactic acid for encapsulation of the anti-HIV drug called Lamivudine; the nanoparticles were found to be around 300 nm and the drug encapsulation efficiency was 75.4%. They were found to be non-toxic to mouse fibroblast cells (L929 cells) [84]. Hydrophilic drugs such as 5-fluorouracil and leucovorin have been encapsulated in chitosan nanoparticles for the treatment of colon cancer. The drug-loaded nanoparticles were in a wide size range of 34–112 nm. The drugs loaded into the nanoparticles initially had a burst release followed by a continuous and constant release of the drugs. Encapsulation efficiency and the drug loading capacity of the drugs were found to be very efficient because of the strong interaction between the biopolymer and the drugs [85]. Chitosan nanoparticles were incorporated into the silk fibroin hydrogel scaffolds for the repair of cartilage defects. The incorporation of tumor growth factor (TGFβ) and bone morphogenic protein (BMP) was carried out to repair the articular defects. Enhanced cell viability, cytocompatibility and chondrogenesis was observed [86]. Curcumin was encapsulated into chitosan nanoparticles and finally incorporated into nanofiber mats containing polycaprolactone and gelatin. The nanoparticles were in the size range of 278 ± 60 nm. The encapsulation efficiency and the drug loading capacity were found to be 93 ± 5% and 4.2 ± 0.2%, respectively. Drug release of the nanocomposite was observed up to 240 h. The cell compatibility of the nanocomposite was studied with the help of human endometrial stem cells (EnSCs), as indicated in Figure 7.

Higher cellular growth was found in the PCL/gelatin/chitosan nanoparticles/curcumin nanofiber mats. An increase in cell adhesion and proliferation of the nanofiber mats was observed at the end of 72 h. The hybrid composite was found to be biocompatible, as observed through MTT assay.

### 3.2. Alginate

**Figure 8:**
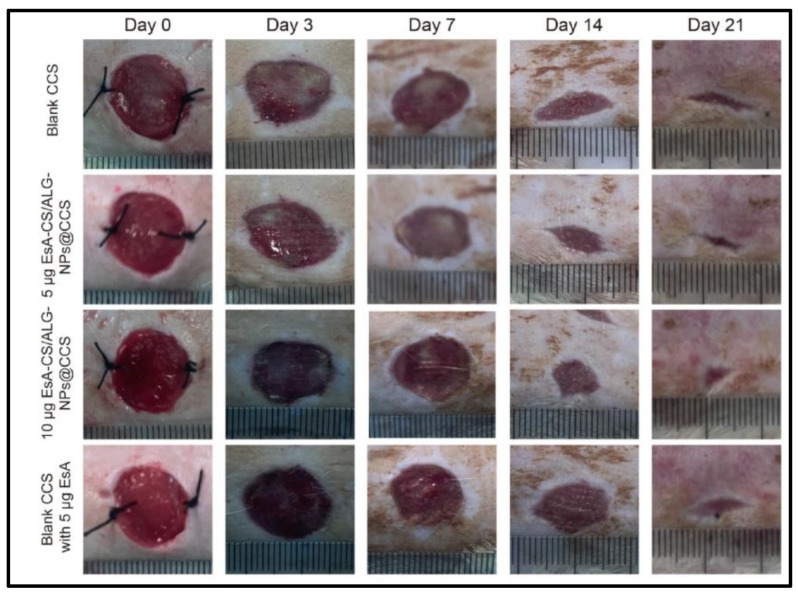
Images of the healing of burn wounds after transplantation with the blank Collagen/chitosan scaffold, 5 μg drug–chitosan/alginate nanoparticles @ collagen/chitosan scaffold, 10 μg drug-chitosan/alginate nanoparticles @collagen/chitosan scaffold, and blank collagen chitosan scaffold with 5 μg drug at days 0, 3, 7, 14, and 21 (Reproduced with permission from [95]; Copyright 2023, American Chemical Society).

Alginate is one of the most important anionic biopolymers obtained from seaweeds such as brown algae. They are linear and are made of units of α-L-guluronic acid and β-D-mannuronic acid linked by 1,4 glycosidic linkages. The presence of carboxyl and hydroxyl groups in their structure facilitates easy modification according to the intended application. It can be transformed into any form, such as nanoparticles, hydrogels, microparticles and porous scaffolds. The ability of alginate to form gels without the addition of any toxic substance at normal conditions has enabled it to be used in a wide variety of therapeutic applications. It is also very easily available, not toxic, and has favorable cell compatibility and biodegradable properties. Alginate nanoparticles are fabricated by means of pre-gelation with calcium; they are widely being explored in the field of tissue engineering, regenerative medicine, wound healing, biosensors, genetic transfection and environmental applications. The nanoparticles show improved biocompatibility, degradation properties and also mucoadhesiveness properties; they are combined with other polymers to modulate their physiochemical, mechanical and biological properties [13,31,88,89,90]. A study reported the use of alginate nanoparticles along with an antibiotic called polymyxin B sulphate to be one of the layers for the biomembrane designed for wound healing. The biomembranes showed low toxicity and were found to be biocompatible with the fibroblast cells; the in vivo analysis showed promising outcomes [91]. Alginate nanoparticles, along with chitosan, were employed for the delivery of the drug called nifedipine. The nanoparticles had an average diameter of 20 to 50 nm. The drug release was found to be pH responsive, i.e., the percentage of the drug varies with respect to the pH. Initial burst release followed by continuous controlled release was observed. Fick’s diffusion was found to be the reason for the drug release [92]. Curcumin diethyl disuccinate was encapsulated in chitosan/alginate nanoparticles for anti-cancer therapy. A sustained release profile of the drug and improved bioavailability was observed. The drug was found to be stable when exposed to digestive fluids. The main mechanism behind the release of the drug was found to be diffusion. It was found that the cellular uptake was enhanced and showed cytotoxicity against the HepG2 cell line [93]. Zohri et al. reported a formulation where chitosan and alginate nanoparticles were used as a non-viral vector for gene delivery applications and optimized using the D-optimal design. The nanoparticles were found to be compatible with cells and a transfection efficiency of 29.9% was observed [94]. One study reported the sustained release of the drug esculentoside from chitosan/alginate nanoparticles that were embedded in a collagen/chitosan scaffold for the treatment of burn wounds. The highest encapsulation efficiency of 78.20% was observed. The composite scaffold showed good anti-inflammatory activity. The in vitro assays showed that M2 macrophages were activated, which promoted quick healing of the burn wounds. The in vivo evaluation of the nanocomposite in the burn wounds also showed promising results (Figure 8).

Drug concentrations in the nanocomposite scaffold showed better healing properties than the blank scaffold. The wound was almost completely healed at the end of day 21 [95].

### 3.3. Starch

Starch is a natural, biodegradable biopolymer obtained from various plants such as potato, wheat, rice or corn, and it is made of amylose and amylopectin. It is widely employed for biomedical applications such as tissue engineering or wound healing. It is easily available since it is the second most abundant biomass present on the earth. The important favorable characteristics that make them a suitable candidate for various applications are that they have swelling characteristics, rheological properties, degradable properties, solubility and biocompatibility. Starch-based nanoparticles are used as fillers with other polymer matrices and help to improve the various physiochemical and mechanical properties. Studies also have reported that starch-based nanoparticles increase encapsulation efficiency. They can be fabricated by a variety of methods such as precipitation, micro fluidization and enzyme hydrolysis, homogenization and emulsification. They have enhanced absorptive capacity and biological penetration rate and are thus employed as carriers to deliver bioactive compounds [10,13,96,97,98,99]. One study reported that CG-1521 was encapsulated in starch nanoparticles for the treatment of breast cancer. Improved therapeutic index and bioavailability were reported due to the presence of nanoparticles. The release rate of the drug was reduced and the cytotoxicity was enhanced towards the MCF-7 cell line. Cell cycle arrest and apoptosis were witnessed in the MCF cell line in in vitro study. The drug delivery of the drug was found to be promising without interfering with the mechanism of drug action [100]. Curcumin was loaded onto starch nanoparticles derived from green bananas. The nanoparticles were found to be about 250 nm in size and the encapsulation efficiency was found to be 80%. More controlled release of curcumin was observed because of the strong hydrogen bond interaction [101]. Starch nanoparticles grafted with folate and biotin for the delivery of Doxorubicin and siRNA. A high amount of cytotoxicity was observed against the A549 cell line (human lung cancer cell line). The lowest amount of cell proliferation was observed and the mechanism behind cellular uptake was found to be either clathirin or caveolae-mediated [102]. A nano-based drug delivery system was designed by using starch nanoparticles conjugated with aptamer loaded with para coumaric acid for the treatment of breast cancer. The nanoparticles were found to be less agglomerated and the particle size was found to be 218.97 ± 3.07 nm. The encapsulation efficiency was found to be 80.30 ± 0.53%. Rapid and burst release of the drug was observed for the initial five hours. Higher cytotoxicity was observed towards MDA-MB-231 cells [103]. Triphala Churna, an ayurvedic drug, was encapsulated in starch nanoparticles for the purpose of releasing various drugs and bioactive compounds. The nanoparticles were in the size range of 282.9 nm. Improved fast drug release was observed at pH 7.4, and enhanced drug encapsulation was observed. The anti-oxidant and anti-bacterial results of the drug-loaded starch nanoparticles showed promising results. The drug showed improved activity and the mechanism of the drug was not altered though it was encapsulated in starch nanoparticles [104]. Methacrylated starch-based nanoparticles have been employed as hydrogels by photopolymerization. Dense and stiff hydrogels that are compatible with human cells were fabricated and reported in a study by Majcher et al. The shear modulus was found to be increased by at least five times [105].

### 3.4. Dextran

Dextran belongs to a family of microbial polysaccharides obtained from lactic acid bacteria (LAB) and their enzymes in the presence of sucrose. This exopolysaccharide is linked by D glucose units majorly by α-1,6 bonds. The physio–chemical properties vary with respect to the strain producing it. The favorable rheological, thermal properties, biocompatibility and biodegradability, enable dextran to be employed in a lot of applications. Dextran has been employed in biomedical applications such as wound healing, tissue engineering, imaging and as drug carriers. The ability of dextran nanoparticles to form a stable backbone has shown promising results to be employed as a nano drug carrier [106,107]. One study reported the use of the anticancer drug doxorubicin encapsulated in carboxymethyl dextran nanoparticles for cancer treatment. The nanoparticles were in the size of 242 nm and had an encapsulation efficiency of greater than 70%. Rapid release of the drug was observed initially. In vitro assays revealed that the fabricated nanoparticles showed higher cytotoxicity towards the SCC7 cancer cell line. A high anti-tumor effect was exhibited from the drug-loaded nanoparticles [108]. A dextran nanoparticle of about 13 nm was crosslinked with Zirconium (Zr-89) to be used as a positron emission tomography (PET) imaging agent for the purpose of imaging macrophages. The half-life was found to be 3.9 h, and they primarily imaged only the tissue macrophages and not the white blood cells. The in vivo imaging results showed that the tumoral uptake was very high and was able to surpass the reticuloendothelial system [109]. Acryloyl crosslinked dextran dialdehyde (ACDD) nanoparticles grafted with glucose oxidase for the fabrication of a pH-responsive insulin delivery system. A controlled release of insulin of 70% was observed in the artificial intestinal fluid conditions for 24 h. In the presence of glucose, the release was found to be 90% under artificial intestinal fluid conditions. The mechanism behind the release of the drug was found to be non-Fickian diffusion [110]. Butzbach et al. reported a study where photosensitizer was encapsulated in spermine and acetyl-modified dextran nanoparticles and grafted with folic acid on the surface that is specifically expressed in the tumor cells. Cellular uptake against He-La KB cells and cytotoxicity induced by light were observed [111]. Another study reported the use of dextran nanoparticles conjugated with acitretin for the treatment of psoriasis-like skin disease. A low dosage of the drug does not induce and side effects. In vitro results showed that keratinocyte proliferation was enhanced. The mechanism behind that was that the STAT-3 phosphorylation was efficiently inhibited [112]. Cerium oxide nanoparticles were coated with dextran for use as a contrast agent in the gastrointestinal tract and bowel diseases. Enhanced imaging in the inflammation sites. No toxicity was observed and was protective against oxidative damage. The oral dose (>97%) was cleared after 24 h [113]. In another study, dextran nanoparticles were cross-linked with colon-specific oligoester that responds to enzymes was fabricated. 5-Fluoro uracil was encapsulated in the dextran nanoparticles for the treatment of cancer. The nanoparticles were in the size range of 237 ± 25 nm. The encapsulation efficiency of the drug was found to be 76%. The drug was found to release only in the presence of the enzyme dextranase. 75% of the drug was released up to 12 h of incubation. The dextran nanoparticles were found to be compatible with the HCT116 colon cancer cell line and were found to be cytotoxic in the presence of the enzyme dextranase [114]. The summary of the polysaccharide based polymeric nanoparticles is given in Table 3.

## 4. Synthetic Biopolymeric Nanoparticles

This type of biopolymer is either obtained by modifying the natural polymers or by chemically synthesized from the monomers in such a way that they do no leave any toxic by product. It can be either obtained from renewable feedstock or from fossil fuels. They are more advantageous than natural polymers and are employed in a variety of applications because of their stability and flexibility. They also facilitate controlled release, non-immunogenic and can be easily cleared from the body. One of the disadvantages of synthetic biopolymers are that they lack cell adhesion sites and chemical modifications are required to improve their property. Some examples of synthetic biopolymers include polycaprolactone (PCL), Polylactic acid (PLA), Polyvinyl alcohol (PVA) and Polyethylene glycol (PEG), which are widely being studied for various biomedical applications. The nanoparticles synthesized out of them have improved properties such as biocompatibility, biodegradability and stability. The higher surface-to-volume ratio enables higher reactivity and a capability to easily modify the functional groups and, thereby, the governing properties [124].

### 4.1. Polycaprolactone Nanoparticles

Polycaprolactone (PCL) is a polymer that is biodegradable and belongs to the family of aliphatic polyesters, and is fabricated by using the polymerization technique using a monomer and an initiator. It is widely used in many biomedical applications such as tissue engineering, wound healing and drug delivery because of its favorable features such as biocompatibility, biodegradability, bioresorbability and rheological properties. PCL is also approved by the Food and Drug Administration (FDA). It is used to deliver multiple drugs and also further includes peptides, proteins and bioactive molecules for various therapeutic applications. The degradation of PCL takes about 2 to 3 years and the by-product is also metabolized by the body [125,126,127,128]. The drugs have been encapsulated in PCL nanoparticles to improve the bioavailability, specificity and the therapeutic index [129]. One study reported the encapsulation of carboplatin in PCL nanoparticles for the purpose of intranasal delivery. The drug-loaded nanoparticles were fabricated by a double solvent evaporation method. They were in the size of 311 ± 4.7 nm. The encapsulation efficiency was found to be 27.95 ± 4.21%. The drug release profile showed a biphasic pattern where there was an initial burst release followed by controlled continuous release. In vitro analysis exhibited an increased cytotoxicity activity against human glioblastoma cells—LN229 cell line. Nasal perfusion studies performed in situ in Wistar rats showed that the absorption capacity of the drug was higher in the case of an encapsulated drug rather than a free drug [130]. PCL, along with Tween 80, was fabricated into nanoparticles and used for loading the drug docetaxel for the purpose of cancer therapy. The nanoparticles were found to be spherical in shape and about 200 nm in diameter. 10% of the drug was encapsulated and nearly 35% got released in a period of 28 days. This combination showed high cellular uptake and exhibited enhanced cytotoxicity towards the C6 glioma cancer cell line [131]. Geranyl cinnamate was encapsulated in PCL nanoparticles to improve its stability and prevent it from thermal degradation. They were fabricated by solvent evaporation method and the particles were found to be spherical with a size of 177.6 nm. The drug-loaded nanoparticles showed stability for 60 days. The drug release occurs only in the presence of an external trigger, such as oil phase or an enzyme to degrade the polymer matrix [132]. Hybrid nanoparticles made of PCL and hydroxyapatite were fabricated to improve osteogenesis. Enhanced cell proliferation and differentiation was observed. A low amount of cell cytotoxicity was reported. Osteogenic markers such as Run x-2 and osteopontin were moderately expressed and sialoprotein was highly expressed after 10 days [133]. Hao et al., reported a study where PCL nanoparticles was grafted with polyethylene glycol and loaded with indocyanine green and 5-fluorouracil for the treatment of skin cancer. This system was integrated with a hyaluronic acid microneedle system. The cell proliferation of A431 and A375 was very well inhibited. The whole system showed an enhanced photothermal effect. Controlled release of the drug and its promising anti-tumor ability was reported [134]. Dorzolamide was encapsulated on to PCL nanoparticles coated with chitosan for ocular drug delivery. The size and the encapsulation efficiency of the nanoparticles were found to be192.38 ± 6.42 nm and 72.48 ± 5.62%. Drug release was found to be a biphasic patter with an initial burst release for 2 h followed by a sustained release for 12 h. Improved permeation rate and mucoadhesive behavior when compared to the control group. Histopathology analysis revealed that they were completely safe to use and did not induce any toxicity [135]. PCL nanoparticles were grafted with polyethylene glycol and were used to load the drug Cabazitaxel for the treatment of colorectal cancer. Improved bioavailability and biocompatibility were reported. Enhanced drug loading capacity, anti-tumor effect and stability were observed [136]. PCL nanoparticles were employed for the simultaneous delivery of two drugs such as Paclitaxel and IR780, for the treatment of ovarian cancer. The nanoparticles were found to have a high drug-loading capacity and the release of the drug was facilitated by the presence of light. They specifically target ovarian cancer cells and accumulated the drug in an in vivo mouse model [137].

### 4.2. Polylactic Acid Nanoparticles

Polylactic acid (PLA) is an FDA-approved biodegradable polymer derived from sources such as corn starch and sugarcane. It is linear and lipophilic in nature and can be obtained from the polycondensation of a monomer called lactic acid. The only degradation product, lactic acid, is either metabolized or eliminated via urine. It is widely used for biomedical applications such as tissue engineering, wound healing, implants and as drug delivery carriers. The disadvantage is that it has poor stability in heat and is very brittle. PLA nanoparticles are fabricated to encapsulate drugs or used as a filler in other polymer matrices. The nano form of PLA improves the stability and reactivity [124]. One study reported the use of PLA nanoparticles to encapsulate quercetin for therapeutic applications. The drug-loaded nanoparticles were prepared by the solvent evaporation method. The drug was loaded to improve the stability, permeation rate and solubility. The size of the particles was found to be 250 ± 68 nm and the encapsulation efficiency to be 40%. The drug release pattern was found to be initially burst followed by sustained release of the drug. The enhanced anti-oxidant activity was reported [138]. Rifampicin was loaded into PLA nanoparticles for the treatment of anti-bacterial actions. They were fabricated by nanoprecipitation method and a two phase drug release was observed. Enhanced antibiotic delivery was reported [139]. Enrique Niza et al., fabricated polyethylene imine coated PLA nanoparticles loaded with a bioactive compound called Carvacrol for enhanced anti-bacterial and anti-oxidant activity. The size and the encapsulation efficiency of the nanoparticles was found to be 100 nm and 30%. Burst release of 15% of the drug followed by sustained drug release at the end of 8 h. Enhanced anti-microbial activity and stability was reported [140]. Berberine is an anti-cancer drug that was loaded into PLA nanoparticles by using coaxial electrospray technique for sustained drug release. The size of the fabricated nanoparticles was found to be 265 nm and the encapsulation efficiency was found to be 81%. High cell cytotoxicity and cellular uptake was reported against HCT116 cell line [141]. PLA nanoparticles was used to encapsulate two drugs daunorubicin and glycyrrhizic acid for simultaneous delivery to treat leukemia. Enhanced encapsulation and loading capacity were observed. Improved drug uptake and further facilitated an increase in apoptosis rate [142]. A novel drug delivery system was designed for the treatment of cancer using PLA nanoparticles loaded with PLX4032 which is an anti-cancer drug. Enhanced loading efficiency and the cancer cells were destroyed. This theranostic device was used for the purpose of cancer treatment [143].

### 4.3. Poly Vinyl Alcohol Nanoparticles

Poly vinyl alcohol (PVA) is a water soluble polyhydroxy polymer that is semi-crystalline and can be obtained from polyvinyl acetate by hydrolysis reaction. They are widely employed for biomedical applications because of their properties such as low cost, compatibility with cells, highly elastic in nature and has tensile strength that matches with that of the articular cartilage. The disadvantages are that it has very less growth of osteoblast cells since it lacks self-adhesion sites [16,144,145]. PVA nanoparticles can be fabricated by techniques such as nanoprecipitation or by emulsion technique. The nanoparticles enable widely in cancer treatment by delivering the drug to the tumor site because of the leaky vessels. PVA nanoparticles aid in improving the bioavailability and the stability of the loaded drug [146]. Zinc oxide/PVA nanoparticles were fabricated by sol–gel method for the purpose of reducing the level of glucose. The nanoparticles were found to be spherical in shape and varying amounts of polyvinyl alcohol had an impact on the photocatalytic activity. The in vivo analysis also showed promising results of reduced glucose levels in rats affected with diabetes [147]. Bovine serum albumin was encapsulated in polyvinyl alcohol nanoparticles for the purpose of delivering peptides. The nanoparticles were fabricated by water in an oil emulsion technique and the diameter of the particles were found to be 675.56 nm. The encapsulation efficiency of the drug was 96.26%. The release of the protein, governed by the diffusion process, was held in a sustained manner that lasted up to 30 h. The stability of the drug was raised when it was loaded onto polymeric nanoparticles [148]. The summary of the synthetic biopolymeric nanoparticles is given in Table 4.

## 5. Fabrication of Biopolymeric Nanoparticles

**Figure 9:**
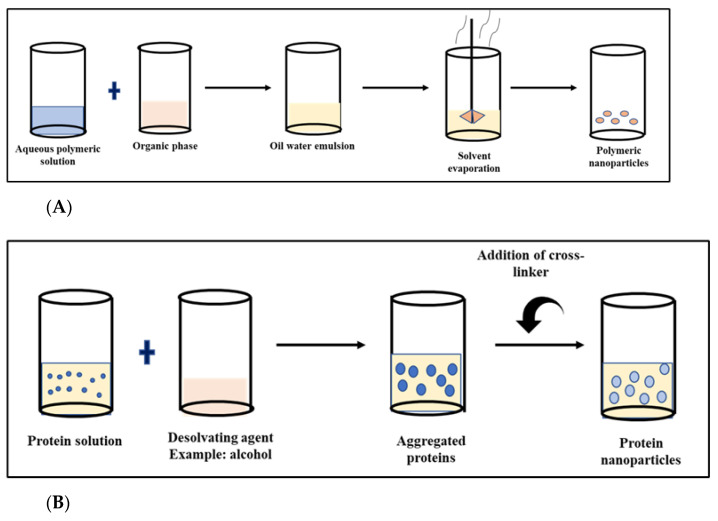

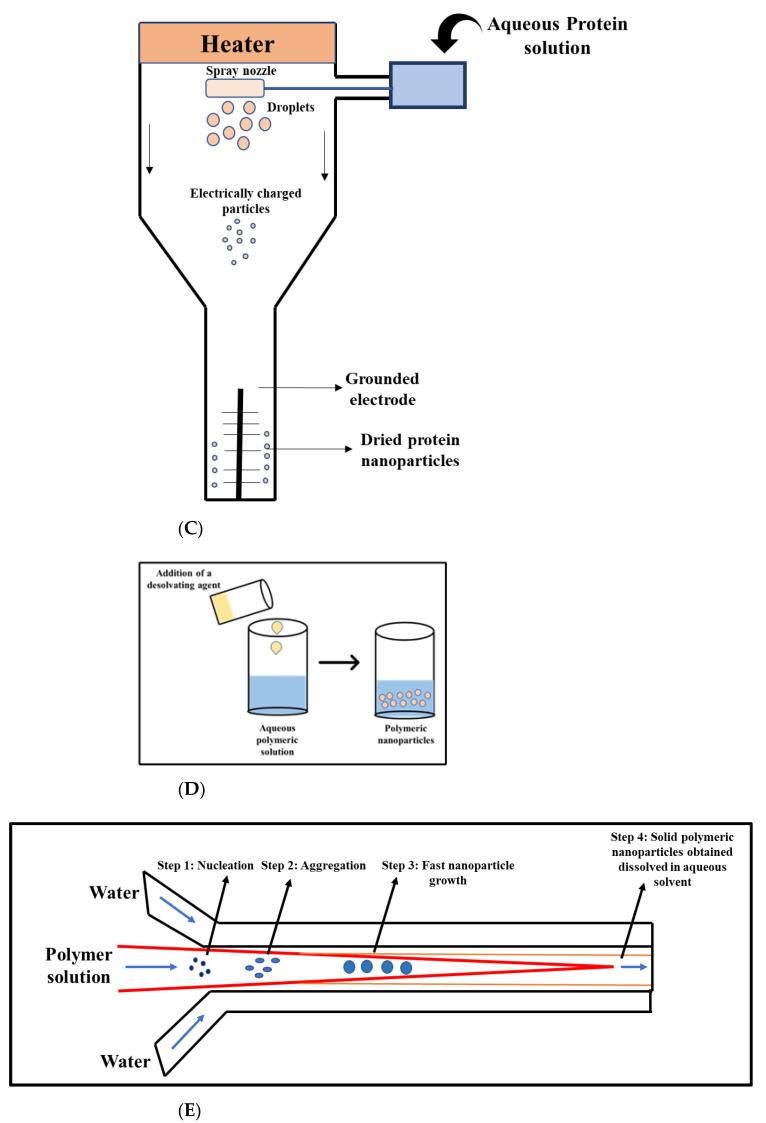
Schematic representation of various methods for biopolymeric nanoparticle fabrication. (**A**) Emulsification, (**B**) phase drying/coacervation, (**C**) spray drying, (**D**) desolvation, (**E**) microfluidics.

The fabrication of the biopolymeric nanoparticles can be either by top-down or bottom-up approaches. The synthesis technique greatly influences the size and the poly-dispersity index of the nanoparticles. An appropriate fabrication process is chosen by considering the required features of the polymeric nanoparticles. Some of the fabrication techniques employed for biopolymeric nanoparticles, such as emulsification, precipitation, coacervation and spray deposition are discussed in the following section [150]. The fabrication strategies of biopolymeric nanoparticles are schematically represented in Figure 9.

### 5.1. Emulsification

This method involves the formation of droplets in the nano range when the aqueous and the organic phase are mixed together in a ratio 2:1. The aqueous phase is usually made of water and a surfactant that is hydrophilic. The organic phase is made of a surfactant that is lipophilic, oils obtained from plants and a solvent that can dissolve in water. They can be either water in water (*W*/*W*) or water in oil emulsion (*W*/*O*) phase. The *W*/*W* phase is employed for fabricating hydrogel-based protein or polysaccharide nanoparticles, and an additional crosslinking, such as treatment using transglutaminase or acidification for the internal phase, can be employed. The *W*/*O* emulsion phase helps to fabricate nanoparticles that are stable with a high yield. The nanoparticles fabricated usually have high drug loading capacity and entrapment efficiency. The solvent in the organic phase can be removed by using the evaporation technique. One of the main disadvantages in this technique is employing and removing the organic solvent since the residues in the end can lead to toxicity [13,150,151].

### 5.2. Desolvation

Desolvation is also known as anti-solvent precipitation and is widely employed for the fabrication of biopolymeric nanoparticles from proteins as well as polysaccharides. Solute precipitation is facilitated when the quality of the solvent employed for dissolving the polymer is reduced. Factors such as pH, the concentration of the cross-linking agent (e.g., glutaraldehyde), and ionic strength can be optimized to control the size of the particles. The solvents include water, supercritical CO_2_ or any organic solvent. The driving force behind the formation of nanoparticles is the imbalance in the interactions between the solute, solvent and anti-solvent. This method is highly preferred since this method does not use high-end equipment and is of low cost [2,13,150,152,153].

### 5.3. Coacervation

This method is similar to the phase separation technique, where there is a separation of the polymer-rich and low-polymer-content phases. The rich polymer phase, known as coacervates, is formed when oppositely charged biopolymers interact that can facilitate the encapsulation of the active ingredient. The solvents usually employed include acetone or ethanol. The fabricated nanoparticles are usually stabilized by adding cross-linking agents such as glutaraldehyde. The factors that have to be noted to control the particle size are the molecular weight and the quantity of the polymer. The main drawbacks of the method are that they have low stability and controlling the size of the biopolymeric nanoparticles is very critical [2,13,154,155].

### 5.4. Spray Deposition

The spray deposition method is also known as electrohydrodynamic atomization, which employs the generation of droplets that are charged as a result of the atomization process by the application of an electrical field. The nanoparticles are dried on the substrate and are strongly bonded. No particular surfactant or template is required for the process. The size of the nanoparticles is altered by the variation in the voltage supply, charge, flow rate, and the distance between the substrate and the needle. This method is highly preferred for the fabrication of biopolymeric nanoparticles, especially drug nanocrystals, since there is no alteration in the biological properties [2,11,13,156].

### 5.5. Microfluidics

Biopolymeric nanoparticles can be synthesized by using microfluidic technology with the aid of micro-reactors that have inner dimensions of less than 1 mm. These microreactors are similar to lab-on-chip devices and are usually made of polymers such as polydimethyl siloxane (PDMS) or glass. They can be either single-phase or multi-phase flow systems. The mechanism behind the formation of polymeric nanoparticles in the microfluidic channel is usually the self-assembly or nanoprecipitation method. The main advantages of this technique being employed for polymeric nanoparticle formation is that they have high reproducibility, low reagent requirement and enhanced control of experimental parameters. The disadvantages of the technique include the design of microfluidic channel being very complex, and there are chances that the nanoparticles can diffuse through the polymeric matrix and cause clogging in the channel [157,158,159].

## 6. Challenges and Future Perspective

Biopolymeric nanoparticles are widely employed for a wide variety of biomedical applications such as tissue engineering, drug delivery systems, imaging and sensor systems for theranostic kits. The properties such as degradability, cell compatibility, improved stiffness and strength makes them very much suitable for various applications [13]. This field is gaining high interest and is reflected in terms of publications by researchers. They are either being patented or in the process of being commercialized. For example, the product Ecosphere^®^ from the company Ecosynthetix, in 2008, developed starch nanoparticles for adhesive purposes owing to its higher surface-to-volume ratio and improved reactivity [99]. Biopolymeric nanoparticles are used in the treatment of cancer owing to their selective tumor-targeting ability. The properties can be tuned appropriately and are supposedly the most suitable candidate for biomedical applications. The high surface-to-volume ratio enhances the molecules’ association and facilitates a high drug encapsulation rate. Surface modification of the biopolymeric nanoparticles can be carried out to improve the circulation time and immunogenic properties. A more efficient drug delivery system can be designed with combined therapeutic and diagnostic for the treatment of various diseases [89]. Some polysaccharide and protein-based biopolymers, such as alginate and bovine serum albumin, have mucoadhesive nature and the small size makes penetration to the target size easier [160]. Focusing on this direction helps to bring in various technological advances in the biomedical sector. One of the main challenges to employing these for biomedical applications is nanoparticle toxicity. There are no standard assessment methods for nanoparticle toxicity. The nanoparticles can accumulate over time in the system and cause side effects. The toxicity differs with the dose and the time of exposure. Though multiple products exist in the market containing nanoparticles, a scientific gap exists since there are no strict regulations. Thus, proper regulatory measures are required when nanoparticles are being dealt with for medical applications. Upscaling the technology or commercialization also plays a key role and remains to be a challenge. Currently, researchers are highly focused on biopolymeric nanoparticles to be employed for biomedical applications with improved efficiency and reduced toxicity [4,13,161,162].

## 7. Conclusions

The use of biopolymeric nanoparticles has proven to be economical, environmentally friendly and promising in the technical aspect for a wide range of applications, especially in the medical domain. A lot of research work is going on employing protein, polysaccharide and synthetic-based biopolymer systems owing to their positive features such as biocompatibility and biodegradability. Nanotechnology is highly blooming in the 21st century and nanoparticles have the innate ability to be modified according to the required application. Biopolymeric nanoparticles are found to be highly stable and show improved biocompatibility, degradation rate and surface reactivity. It is very critical and important to produce biopolymeric nanoparticles of favorable size and properties to be employed in fabricating novel drug delivery systems for sustained drug release. The choice of the nanoparticle depends on the application and the properties can be tuned according to the intended application. Surface modification of the biopolymeric nanoparticles aids in the enhancement of the circulation time and prevents immunogenic reactions. This review focused on the various biopolymeric nanoparticles fabricated for biomedical applications such as drug delivery, imaging and tissue engineering. The important fabrication techniques, along with the challenges and the future perspective in this domain, were also discussed. The initial stage for the development of the biopolymeric nanoparticles requires expensive instruments and up-scaling the technology is also challenging. Thus, future researchers should focus on this and on ways to make sure that the nanoparticles do not induce bioaccumulation in the human system. It is also necessary to develop nanoparticles with enhanced efficacy. A deep and clear understanding of nanoparticle–immune system interaction and the elimination from the human system is an important concern and must be addressed in the future.

## Figures and Tables

**Table 1:** Summary of advantages and disadvantages of various biopolymeric nanoparticles.

Polymer	Advantages	Disadvantages	Reference
Albumin	Highly abundant, biodegradable, biocompatible, non-cytotoxic.	Immunogenic effects, very expensive, lack of efficacy.	[18,19]
Gelatin	Enhanced cell adhesion, proliferation and cell infiltration in the scaffolds, good stability and biodegradability, osteoconductive, non-immunogenic	Low stability in normal physiological conditions, poor bioactivity, brittle, fast degradation rate under physiological conditions	[16,20]
Silk fibroin	Biocompatible, osteoconductive, improves cell migration and angiogenesis, good elastic properties, moderate degradation rate.	Low mechanical strength, degradation of silk releases by-products that can cause immunogenic reactions, inability to induce osteogenesis.	[16,20,21]
Collagen	Low immunogenicity, enhanced permeability properties, excellent cell adhesion, proliferation and differentiation properties, biodegradable, biocompatible.	Low mechanical strength, low structure stability, variability in different collagen sources.	[16,20,22]
Chitosan	Mucoadhesive nature, enhanced biocompatibility, osteoconductive, non-toxic, promotes cell adhesion, hemostatic potential, biodegradable, anti-bacterial activity.	In vivo degradation rate is very high, low mechanical strength, cross-linkers are required to maintain stability, solubility is less and viscosity is high at neutral pH, control of nanoparticle size is difficult.	[16,20,23]
Alginate	Biocompatible, biodegradable, cell compatible, gel-forming capability, low immunogenicity, mimics the extracellular matrix, low cost, ability of encapsulation.	Low mechanical properties, degradation is questionable sometimes, poor cell adhesion, sterilization is difficult.	[16,20,24]
Starch	Biodegradable, low cost, biocompatible, easily available, good cell adhesion.	Very high viscosity, low Mechanical properties, fragile, stability issues, water uptake is very high, modifying chemically can release toxic by-products.	[16,25]
Dextran	Biocompatible, anti-thrombotic property, good water solubility, functionalization can be carried out easily.	High cost, non-availability, very high permeability, encapsulated drugs are released very fast.	[26,27]
Poly- caprolactone	Compatible with cells, non-toxic, cell proliferation and angiogenesis can be controlled, good mechanical properties, improved cellular proliferation.	Bioactivity is less, poor cellular adhesion due to hydrophobic surface, use of toxic solvents.	[16]
Polyvinyl alcohol	Biocompatible, good elastic nature, water-soluble polymer, good tensile strength, improved flexibility, stability to various temperatures, low cost.	Lacks cell adhesion property, in growth of bone cells is significantly less, very high water uptake.	[16,28,29]
Polylactic acid	Biocompatible, cell compatible, degradation rate is good, by-products are non-toxic, properties can be easily tailored, eco-friendly.	Lack of cell adhesion and proliferation property, expensive, brittle (elongation at break is less than 10%), chemically inert.	[16,30]

**Table 2:** Summary of protein-based biopolymeric nanoparticles.

Protein	Overall Composition	Application	Key Findings of the Study	Reference
Albumin	Human serum albumin + ibrutinib and hydroxychloroquine (nanoparticles)	Co-drug delivery system for treatment of glioma	Improved bioavailability Prolonged survival time in in vivo treated mice High cytotoxicity against C6 cells	[36]
Albumin	Bovine serum albumin + abaloparatide + aspirin + polycaprolactone + hydroxyapatite (nanofibrous scaffold)	Bone regeneration	Improved degradation rate Slow drug release Enhanced compatibility Improved bone regeneration	[37]
Albumin	Human serum albumin (HSA) + indocyanine green (ICG) + paclitaxel (PTX) + hyaluronic acid (nanoparticles)	Image-guided drug delivery	Efficient drug release in the tumor environment Efficient anti-cancer activity	[38]
Albumin	Bovine serum albumin + carnosic acid	Anti-tumor activity of breast cancer and colon cancer.	Enhanced loading activity Improved release profile of the drug Enhanced anti-tumor activity Upregulation of *GCLC* gene and downregulation of *BCL-2* and *COX-2* gene.	[39]
Albumin	Bovine serum albumin + silymarin + curcumin + chitosan	Muco-inhalable drug delivery system	Significant reduction of interleukin-6 and c-reactive protein Efficient anti-viral activity in in vitro COVID-19 experiment	[71]
Albumin	Bovine serum albumin + poly-L-lysine + graphene oxide	Bone regeneration	Controlled release of BMP-2 (14 days) Improved matrix mineralization Enhanced Alkaline phosphatase (ALP) activity	[72]
Gelatin	Gelatin + concanavalin-A + cisplatin	Drug delivery for cancer therapy	Enhanced cellular uptake of nanoparticles Enhanced reactive oxygen species and apoptosis in cancer cells	[73]
Gelatin	Gelatin methacrylol nanoparticles + rhodamine	Cell imaging	Improved cell viability and cell proliferation in vitro Superior cell compatibility Enhanced cellular uptake Improved fluorescent properties	[74]
Gelatin	Amino cellulose + polycaprolactone + gelatin nanoparticles	Rheumatoid arthritis	Reduction in swelling and inflammation in rats. Maintaining cartilage and bone tissue architecture. Reduction of inflammatory markers	[75]
Gelatin	Gelatin + indocyanine + doxorubicin	Breast cancer treatment	Improved drug release Suppressed the tumor growth in vivo Enhanced degradation of matrix metalloproteinase-2	[76]
Gelatin	Polyethylene glycol grafted gelatin nanoparticles + doxorubicin + betanin	Cancer therapy	Enhanced cellular uptake Cell apoptosis induced in MCF cells; Controlled drug release observed	[43]
Silk fibroin	Curcumin + silk fibroin nanoparticles	Cancer therapy	Enhance anti-tumor activity Improved anti-oxidant activity Curcumin was found to be fluorescent when encapsulated	[52]
Silk fibroin	Silk fibroin + sodium alginate + silk fibroin nanoparticles (scaffold)	Wound healing	Improved cell adhesion Enhanced hemostasis Improved platelet adhesion Excellent biocompatibility and improved cell adhesion and proliferation	[53]
Silk fibroin	Silk fibroin + simvastatin (nanoparticles)	Bone regeneration	Sustained release profile Improved ALP production Enhanced production of osteoblast cells	[54]
Silk fibroin	Silk fibroin + 5 fluorouracil (nanoparticles)	Drug delivery	Improved loading efficiency Slower release of the drug	[55]
Silk fibroin	Silk fibroin nanoparticles + PX478 + doxorubicin	Reverse multi-drug resistance	Increased cellular uptake Downregulation of genes-MDR1, VEGF and GLUT-1	[56]
Silk fibroin	Silk fibroin + tamoxifen (nanoparticles)	Breast cancer	The particle size was found to be 186.1 nm Encapsulation efficiency was found to be 79.08% Biphasic release profile was observed	[77]
Collagen	Collagen + estradiol–hemihydrate	Transdermal drug delivery	Enhanced drug loading capacity Increased sustained drug release Improved drug absorption	[64]
Elastin	Elastin-like polymeric nanoparticles + bone morphogenic protein	Drug delivery system	Improved encapsulation efficiency Compatible with C2C12 cells	[69]
Elastin	α-elastin + methoxy polyethylene glycol + BSA/Insulin	Protein delivery	Encapsulation at low temperatures with simple mixing Sustained release for 72 h The nanoparticles are of normal size distribution	[70]
Elastin	Elastin-like recombinamers + docetaxel + RGD peptide	Drug delivery system	High yield of 70% Monodispersed nanoparticles-40 nm Very much effective against breast cancer cell line	[78]

**Table 3:** Summary of polysaccharide-based polymeric nanoparticles.

Polysaccharide	Overall Composition	Application	Key Findings of the Study	References
Chitosan	Chitosan + polylactic acid + lamivudine	Drug delivery	Drug release was found to be higher when higher percentage was loading The nanoparticles were found to be non-toxic to the L929 cell line The degradation rate increases with respect to pH	[84]
Chitosan	Chitosan + 5-fluorouracil and leucovorin	Drug delivery	Improved encapsulation efficiency and drug loading capacity Release profile can be modulated by changing the parameters	[85]
Chitosan	Chitosan + ellagic acid	Oral cancer therapy	Particle size was found to be 176 nm; Encapsulation efficiency was found to be 94 ± 1.03%. Sustained release of the drug was observed. Cytotoxicity was observed in KB cell line	[115]
Chitosan	Chitosan + tetracycline+ gentamycin + ciproflaxin	Drug delivery	Superior antibacterial properties; improved physiochemical and mechanical properties; greater penetration of nanoparticles observed in the fiber	[116]
Chitosan	Chitosan + 5-fluorouracil	Drug delivery	Negative binding energy makes it energetically suitable; high drug loading capacity; reduced toxicity and increased reactivity	[117]
Chitosan	Chitosan + dexamethasone	Drug delivery	Particle size ranged from 277 to 289 nm; Drug release increased up to 8 h and was constant upto 48 h. Mild cytotoxicity was observed against L929, HCEC and RAW 264.7 cells. Effective anti-inflammatory activity against RAW macrophages	[118]
Chitosan	Chitosan + sodium alginate + polyvinyl alcohol + rosuvastatin	Drug delivery	Enhanced mechanical properties of the hydrogel film. The size of the nanoparticles ranged between 100–150 nm. Encapsulated drug was released within 24 h. High cell viability of fibroblast cells observed after 72 h of incubation	[119]
Alginate	Alginate + rifampicin/isoniazid/pyrazinamide/ethambutol	Anti-tuberculosis drug carrier	High drug encapsulation ranging from 70 to 90%. Improved bioavailability of the drugs Promising in vivo results	[120]
Alginate	Chitosan + alginate nanoparticles + curcumin diethyl disuccinate	Drug delivery	Enhanced stability; good bioavailability; improved cellular uptake; cytotoxicity against Hep G2 cell line.	[93]
Alginate	Chitosan oligosaccharide + alginate nanoparticles + astaxanthin	Drug delivery	Encapsulation efficiency and drug loading capacity were found to be 71.3% and 6.9%. Exhibited stability in acidic, alkaline and ultraviolet light. Sustained drug release was observed. Improved bioavailability and anti-oxidant activity.	[121]
Alginate	Chitosan + alginate nanoparticles	Gene delivery	Particle size of 111 nm; no toxicity observed; transfection efficiency of 29.9%	[94]
Alginate	Chitosan + alginate nanoparticles + esculentoside	Wound healing	Enhanced healing rate; improved anti-inflammatory activity; Sustained drug release rate	[95]
Starch	Starch nanoparticles + citric acid (nanocomposite)	-	The size of the nanoparticles ranged from 50 to 100 nm. Improved storage modulus and glass transition temperature. Decrease in water vapor permeability	[122]
Starch	Starch + CG-1521	Breast cancer treatment	Slow release of the drug; Improved cytotoxicity towards MCF-7 cell line. Cell cycle arrest was induced and apoptosis was seen in MCF-7 cells	[100]
Starch	Starch nanoparticles + curcumin	Drug delivery	Enhanced encapsulation efficiency (80%) Controlled release observed	[101]
Starch	Starch nanoparticles + doxorubicin + siRNA	Cancer therapy	Low cell proliferation; enhanced cytotoxicity against A549 cell line; decreased expression of IGFR 1 protein	[102]
Starch	Starch nanoparticles + para coumaric acid	Breast cancer	Increased cytotoxicity towards MDA-MB-231 cells; burst release observed initially; enhanced encapsulation efficiency	[103]
Starch	Starch nanoparticles + triphala churna	Drug delivery system	Enhanced encapsulation efficiency Improved anti-bacterial and anti-oxidant activity; initial drug release was found to be very fast	[104]
Dextran	Dextran nanoparticles + doxorubicin	Cancer therapy	Enhanced anti-tumor effect; high cytotoxicity towards SCC7 cancer cell line; improved encapsulation efficiency	[108]
Dextran	Zirconium-89 labeled dextran nanoparticles	In vivo imaging	Enhanced tumor uptake; half-life of 3.9 h. Targets only tissue macrophages	[109]
Dextran	Dextran nanoparticles + glucose oxidase	Insulin delivery	Controlled release of insulin -90% under artificial intestinal fluid conditions; mechanism—Non-Fickian diffusion	[110]
Dextran	Dextran nanoparticles + acitretin	Treatment of psoriasis skin disease	Average size of 100 nm; sustained release of 80%. Enhanced proliferation level of keratinocytes; improved inhibition of STAT-3 phosphorylation	[112]
Dextran	Carboxymethyl dextran nanoparticles + Cy-5 labeling	Retinoblastoma	Enhanced ocular bioavailability; more affinity toward ocular tumor	[123]
Dextran	Dextran nanoparticles + Cerium oxide nanoparticles	CT contrast imaging agent	Oxidative stress protection; no toxicity observed; majority of the drug released in 24 h	[113]

**Table 4:** Summary of synthetic bio polymeric nanoparticles.

Synthetic Biopolymer	Overall Composition	Application	Key Findings of the Study	References
Polycaprolactone	Polycaprolactone nanoparticles + carboplatin	Intra nasal delivery	Size- 311.6 ± 4.7 nm; Biphasic pattern of drug release-initial burst release followed by slow and controlled release. Cytotoxic towards human glioblastoma cell line. Better nasal absorption than free drug	[130]
Polycaprolactone	Polycaprolactone + Tween 80 + docetaxel	Cancer therapy	Enhanced cellular uptake; Improved cytotoxicity against C6 glioma cells; 35% of the drug released in 28 days.	[131]
Polycaprolactone	Polycaprolactone nanoparticles + paclitaxel	Cancer therapy	Enhanced encapsulation efficiency; the size was found to be 140 nm. Cell viability reduced against SKOV-3 cell line	[132]
Polycaprolactone	Polycaprolactone nanoparticles + α-tocopherol	-	Decrease in encapsulation efficiency, particle size when the ultrasonication time was increased.	[149]
Polycaprolactone	Polycaprolactone + hydroxyapatite	Bone tissue engineering	Enhanced cell proliferation and differentiation; Moderate expression of markers such as Runx-2 and osteopontin. High expression of sialoprotein at the end of 10 days.	[133]
Polycaprolactone	Polycaprolactone + chitosan + dorzolamide	Ocular drug delivery	Biphasic pattern of drug release; Enhanced drug permeation rate; Improved mucoadhesion; It was found to be non-cytotoxic and safe to use.	[135]
Polylactic acid	Polylactic acid + quercitrin	Therapeutic effect	Size- 250 ± 68 nm; encapsulation efficiency −40%; drug release -burst release followed by sustained release. Enhanced anti-oxidant activity.	[138]
Polylactic acid	Polylactic acid + rifampicin	Antibacterial activity	Biphasic drug release; Improved antibiotic efficiency	[139]
Polylactic acid	Polylactic acid + polyethylene imine coating + carvacrol	Anti-oxidant and Antibacterial activity	Enhanced anti-oxidant and antimicrobial activity. Improved stability rate	[140]
Polylactic acid	Polylactic acid + berberine	Drug delivery system	Technique: coaxial electrospray; high cellular uptake; cell cytotoxicity against HCT116 cell line; slow release profile of the drug was reported	[141]
Polylactic acid	Polylactic acid + daunorubicin + glycyrrhizic acid	Leukemia	Inhibited leukemia cells; enhanced drug uptake; improved apoptosis rate	[142]
Polyvinyl alcohol	ZnO + polyvinyl alcohol nanoparticles	Treatment of diabetes	Exhibited photocatalytic activity In vivo analysis reported lower glucose level	[147]
Polyvinyl alcohol	Bovine serum albumin + polyvinyl alcohol nanoparticles	Delivery of proteins	High drug loading capability; drug release up to 30 h controlled by diffusion process; Enhanced stability of the loaded drug	[148]

## Data Availability

Data sharing is not available.

## References

[1] Silva G.A. (2004). Introduction to nanotechnology and its applications to medicine. Surg. Neurol..

[2] Sundar S., Kundu J., Kundu S.C. (2010). Biopolymeric nanoparticles. Sci. Technol. Adv. Mater..

[3] Couvreur P. (2013). Nanoparticles in drug delivery: Past, present and future. Adv. Drug Deliv. Rev..

[4] Hasan A., Morshed M., Memic A., Hassan S., Webster T.J., Marei H. (2018). Nanoparticles in tissue engineering: Applications, challenges and prospects. Int. J. Nanomed..

[5] Luo X., Morrin A., Killard A., Smyth M.R. (2006). Application of Nanoparticles in Electrochemical Sensors and Biosensors. Electroanalysis.

[6] Tian H., Chen J., Chen X. (2013). Nanoparticles for Gene Delivery. Small.

[7] Kolosnjaj-Tabi J., Wilhelm C., Clément O., Gazeau F. (2013). Cell labeling with magnetic nanoparticles: Opportunity for magnetic cell imaging and cell manipulation. J. Nanobiotechnology.

[8] Bhirde A., Xie J., Swierczewska M., Chen X. (2011). Nanoparticles for cell labeling. Nanoscale.

[9] Issa B., Obaidat I.M., Albiss B.A., Haik Y. (2013). Magnetic Nanoparticles: Surface Effects and Properties Related to Biomedicine Applications. Int. J. Mol. Sci..

[10] Nitta S.K., Numata K. (2013). Biopolymer-Based Nanoparticles for Drug/Gene Delivery and Tissue Engineering. Int. J. Mol. Sci..

[11] Vodyashkin A.A., Kezimana P., Vetcher A.A., Stanishevskiy Y.M. (2022). Biopolymeric Nanoparticles–Multifunctional Materials of the Future. Polymers.

[12] Yadav P., Yadav H., Shah V.G., Shah G., Dhaka G. (2015). Biomedical Biopolymers, their Origin and Evolution in Biomedical Sciences: A Systematic Review. J. Clin. Diagn. Res..

[13] Verma M.L., Dhanya B., Sukriti, Rani V., Thakur M., Jeslin J., Kushwaha R. (2020). Carbohydrate and protein based biopolymeric nanoparticles: Current status and biotechnological applications. Int. J. Biol. Macromol..

[14] Elzoghby A.O., Samy W.M., Elgindy N.A. (2012). Protein-based nanocarriers as promising drug and gene delivery systems. J. Control. Release.

[15] Mizrahi S., Peer D. (2012). Polysaccharides as building blocks for nanotherapeutics. Chem. Soc. Rev..

[16] Reddy M.S.B., Ponnamma D., Choudhary R., Sadasivuni K.K. (2021). A Comparative Review of Natural and Synthetic Biopolymer Composite Scaffolds. Polymers.

[17] Yoha K.S., Priyadarshini S.R., Moses J.A., Anandharamakrishnan C. (2020). Surface Modification of Bio-polymeric Nanoparticles and Its Applications. Advanced Structured Materials.

[18] Meng R., Zhu H., Wang Z., Hao S., Wang B. (2022). Preparation of Drug-Loaded Albumin Nanoparticles and Its Application in Cancer Therapy. J. Nanomater..

[19] Pulimood T.B., Park G.R. (2000). Debate: Albumin administration should be avoided in the critically ill. Crit. Care.

[20] Sergi R., Bellucci D., Cannillo V. (2020). A Review of Bioactive Glass/Natural Polymer Composites: State of the Art. Materials.

[21] Liu J., Ge X., Liu L., Xu W., Shao R. (2022). Challenges and opportunities of silk protein hydrogels in biomedical applications. Mater. Adv..

[22] Dong C., Lv Y. (2016). Application of Collagen Scaffold in Tissue Engineering: Recent Advances and New Perspectives. Polymers.

[23] Garg U., Chauhan S., Nagaich U., Jain N. (2019). Current Advances in Chitosan Nanoparticles Based Drug Delivery and Targeting. Adv. Pharm. Bull..

[24] Gheorghita Puscaselu R., Lobiuc A., Dimian M., Covasa M. (2020). Alginate: From Food Industry to Biomedical Applications and Management of Metabolic Disorders. Polymers.

[25] Zarski A., Bajer K., Kapuśniak J. (2021). Review of the Most Important Methods of Improving the Processing Properties of Starch toward Non-Food Applications. Polymers.

[26] Das M., Shukla F., Thakore S. (2021). Carbohydrate-derived functionalized nanomaterials for drug delivery and environment remediation. Handbook of Functionalized Nanomaterials.

[27] Varghese S.A., Rangappa S.M., Siengchin S., Parameswaranpillai J. (2020). Natural polymers and the hydrogels prepared from them. Hydrogels Based on Natural Polymers.

[28] Gaaz T.S., Sulong A.B., Akhtar M.N., Kadhum A.A.H., Mohamad A.B., Al-Amiery A.A. (2015). Properties and Applications of Polyvinyl Alcohol, Halloysite Nanotubes and Their Nanocomposites. Molecules.

[29] Jain N., Singh V.K., Chauhan S. (2017). A review on mechanical and water absorption properties of polyvinyl alcohol based composites/films. J. Mech. Behav. Mater..

[30] Casalini T., Rossi F., Castrovinci A., Perale G. (2019). A Perspective on Polylactic Acid-Based Polymers Use for Nanoparticles Synthesis and Applications. Front. Bioeng. Biotechnol..

[31] Wong K.H., Lu A., Chen X., Yang Z. (2020). Natural Ingredient-Based Polymeric Nanoparticles for Cancer Treatment. Molecules.

[32] DeFrates K., Markiewicz T., Gallo P., Rack A., Weyhmiller A., Jarmusik B., Hu X. (2018). Protein Polymer-Based Nanoparticles: Fabrication and Medical Applications. Int. J. Mol. Sci..

[33] Mahmoudi M., Lynch I., Ejtehadi M.R., Monopoli M.P., Bombelli F.B., Laurent S. (2011). Protein−Nanoparticle Interactions: Opportunities and Challenges. Chem. Rev..

[34] Stein N.C., Mulac D., Fabian J., Herrmann F.C., Langer K. (2020). Nanoparticle albumin-bound mTHPC for photodynamic therapy: Preparation and comprehensive characterization of a promising drug delivery system. Int. J. Pharm..

[35] Hornok V. (2021). Serum Albumin Nanoparticles: Problems and Prospects. Polymers.

[36] Yang Z., Du Y., Lei L., Xia X., Wang X., Tong F., Li Y., Gao H. (2023). Co-delivery of ibrutinib and hydroxychloroquine by albumin nanoparticles for enhanced chemotherapy of glioma. Int. J. Pharm..

[37] Lin P., Zhang W., Chen D., Yang Y., Sun T., Chen H., Zhang J. (2022). Electrospun nanofibers containing chitosan-stabilized bovine serum albumin nanoparticles for bone regeneration. Colloids Surf. B Biointerfaces.

[38] Thangavel K., Lakshmikuttyamma A., Thangavel C., Shoyele S.A. (2022). CD44-targeted, indocyanine green-paclitaxel-loaded human serum albumin nanoparticles for potential image-guided drug delivery. Colloids Surf. B Biointerfaces.

[39] Khella K.F., El Maksoud A.I.A., Hassan A., Abdel-Ghany S.E., Elsanhoty R.M., Aladhadh M.A., Abdel-Hakeem M.A. (2022). Carnosic Acid Encapsulated in Albumin Nanoparticles Induces Apoptosis in Breast and Colorectal Cancer Cells. Molecules.

[40] El Tokhy S.S., Elgizawy S.A., Osman M.A., Goda A.E., Unsworth L.D. (2022). Tailoring dexamethasone loaded albumin nanoparticles: A full factorial design with enhanced anti-inflammatory activity In vivo. J. Drug Deliv. Sci. Technol..

[41] Yasmin R., Shah M., Khan S.A., Ali R. (2017). Gelatin nanoparticles: A potential candidate for medical applications. Nanotechnol. Rev..

[42] Elzoghby A.O. (2013). Gelatin-based nanoparticles as drug and gene delivery systems: Reviewing three decades of research. J. Control. Release.

[43] Amjadi S., Hamishehkar H., Ghorbani M. (2019). A novel smart PEGylated gelatin nanoparticle for co-delivery of doxorubicin and betanin: A strategy for enhancing the therapeutic efficacy of chemotherapy. Mater. Sci. Eng. C Mater. Biol. Appl..

[44] Yang X.-J., Wang F.-Q., Lu C.-B., Zou J.-W., Hu J.-B., Yang Z., Sang H.-X., Zhang Y. (2020). Modulation of bone formation and resorption using a novel zoledronic acid loaded gelatin nanoparticles integrated porous titanium scaffold: An in vitro and in vivo study. Biomed. Mater..

[45] El-Sayed N., Trouillet V., Clasen A., Jung G., Hollemeyer K., Schneider M. (2019). NIR-Emitting Gold Nanoclusters–Modified Gelatin Nanoparticles as a Bioimaging Agent in Tissue. Adv. Healthc. Mater..

[46] Ahmad A., Ansari M., Mishra R.K., Kumar A., Vyawahare A., Verma R.K., Raza S.S., Khan R. (2021). Enteric-coated gelatin nanoparticles mediated oral delivery of 5-aminosalicylic acid alleviates severity of DSS-induced ulcerative colitis. Mater. Sci. Eng. C.

[47] Zhao Z., Chen A., Li Y., Hu J., Liu X., Li J., Zhang Y., Li G., Zheng Z. (2012). Fabrication of silk fibroin nanoparticles for controlled drug delivery. J. Nanoparticle Res..

[48] Lujerdean C., Baci G.-M., Cucu A.-A., Dezmirean D.S. (2022). The Contribution of Silk Fibroin in Biomedical Engineering. Insects.

[49] Kundu J., Chung Y.-I., Kim Y.H., Tae G., Kundu S.C. (2010). Silk fibroin nanoparticles for cellular uptake and control release. Int. J. Pharm..

[50] Mathur A.B., Gupta V. (2010). Silk fibroin-derived nanoparticles for biomedical applications. Nanomedicine.

[51] Xu Z., Shi L., Yang M., Zhu L. (2019). Preparation and biomedical applications of silk fibroin-nanoparticles composites with enhanced properties—A review. Mater. Sci. Eng. C.

[52] Montalbán M.G., Coburn J.M., Lozano-Pérez A.A., Cenis J.L., Víllora G., Kaplan D.L. (2018). Production of Curcumin-Loaded Silk Fibroin Nanoparticles for Cancer Therapy. Nanomaterials.

[53] Shen Y., Wang X., Li B., Guo Y., Dong K. (2022). Development of silk fibroin-sodium alginate scaffold loaded silk fibroin nanoparticles for hemostasis and cell adhesion. Int. J. Biol. Macromol..

[54] Fatemeh M., Hamidreza M., Ali S.M., Shahrokh S., Mehdi F. (2022). Fabri-cation of Silk Scaffold Containing Simvastatin-Loaded Silk Fibroin Nanoparticles for Regenerating Bone Defects. Iran. Biomed. J..

[55] Rahmani H., Fattahi A., Sadrjavadi K., Khaledian S., Shokoohinia Y. (2019). Preparation and Characterization of Silk Fibroin Nanoparticles as a Potential Drug Delivery System for 5-Fluorouracil. Adv. Pharm. Bull..

[56] Li Z., Cheng G., Zhang Q., Wu W., Zhang Y., Wu B., Liu Z., Tong X., Xiao B., Cheng L. (2022). PX478-loaded silk fibroin nanoparticles reverse multidrug resistance by inhibiting the hypoxia-inducible factor. Int. J. Biol. Macromol..

[57] Nidhin M., Vedhanayagam M., Sangeetha S., Kiran M.S., Nazeer S.S., Jayasree R.S., Sreeram K.J., Nair B.U. (2014). Fluorescent nanonetworks: A novel bioalley for collagen scaffolds and Tissue Engineering. Sci. Rep..

[58] Liu C.Z., Czernuszka J.T. (2007). Development of biodegradable scaffolds for tissue engineering: A perspective on emerging technology. Mater. Sci. Technol..

[59] Gómez-Guillén M., Giménez B., López-Caballero M., Montero M. (2011). Functional and bioactive properties of collagen and gelatin from alternative sources: A review. Food Hydrocoll..

[60] Grigore M.E. (2017). Hydrogels for Cardiac Tissue Repair and Regeneration. J. Cardiovasc. Med. Cardiol..

[61] Lo S., Fauzi M. (2021). Current Update of Collagen Nanomaterials—Fabrication, Characterisation and Its Applications: A Review. Pharmaceutics.

[62] Arun A., Malrautu P., Laha A., Luo H., Ramakrishna S. (2021). Collagen Nanoparticles in Drug Delivery Systems and Tissue Engineering. Appl. Sci..

[63] Alarcon E.I., Udekwu K., Skog M., Pacioni N.L., Stamplecoskie K.G., González-Béjar M., Polisetti N., Wickham A., Richter-Dahlfors A., Griffith M. (2012). The biocompatibility and antibacterial properties of collagen-stabilized, photochemically prepared silver nanoparticles. Biomaterials.

[64] Nicklas M., Schatton W., Heinemann S., Hanke T., Kreuter J. (2009). Preparation and characterization of marine sponge collagen nanoparticles and employment for the transdermal delivery of 17β-estradiol-hemihydrate. Drug Dev. Ind. Pharm..

[65] Foster J.A., Mecham R., Imberman M., Faris B., Franzblau C. (1977). A High Molecular Weight Species of Soluble Elastin-Proelastin. Adv. Exp. Med. Biol..

[66] Seyoung H., Wook C.D., Nam K.H., Gwon P.C. (2020). Protein-Based Nanoparticles as Drug Delivery Sys-tems. Pharmaceutics.

[67] Almine J.F., Bax D.V., Mithieux S.M., Nivison-Smith L., Rnjak J., Waterhouse A., Wise S.G., Weiss A.S. (2010). Elastin-based materials. Chem. Soc. Rev..

[68] Wu Y., Mackay J.A., McDaniel J.R., Chilkoti A., Clark R.L. (2008). Fabrication of Elastin-Like Polypeptide Nanoparticles for Drug Delivery by Electrospraying. Biomacromolecules.

[69] Machado R., Bessa P.C., Reis R.L., Rodriguez-Cabello J.C., Casal M. (2012). Elastin-Based Nanoparticles for Delivery of Bone Morphogenetic Proteins. Nanoparticles in Biology and Medicine.

[70] Kim J.D., Jung Y.J., Woo C.H., Choi Y.C., Choi J.S., Cho Y.W. (2017). Thermo-responsive human α-elastin self-assembled nanoparticles for protein delivery. Colloids Surf. B Biointerfaces.

[71] Hanafy N.A., El-Kemary M.A. (2022). Silymarin/curcumin loaded albumin nanoparticles coated by chitosan as muco-inhalable delivery system observing anti-inflammatory and anti COVID-19 characterizations in oleic acid triggered lung injury and in vitro COVID-19 experiment. Int. J. Biol. Macromol..

[72] Liu F., Xue L., Xu L., Liu J., Xie C., Chen C., Liu Y. (2022). Preparation and characterization of bovine serum albumin nanoparticles modified by Poly-l-lysine functionalized graphene oxide for BMP-2 delivery. Mater. Des..

[73] Vaghasiya K., Ray E., Singh R., Jadhav K., Sharma A., Khan R., Katare O.P., Verma R.K. (2021). Efficient, enzyme responsive and tumor receptor targeting gelatin nanoparticles decorated with concanavalin-A for site-specific and controlled drug delivery for cancer therapy. Mater. Sci. Eng. C.

[74] Xue Y., Lee J., Kim H.-J., Cho H.-J., Zhou X., Liu Y., Tebon P., Hoffman T., Qu M., Ling H. (2020). Rhodamine Conjugated Gelatin Methacryloyl Nanoparticles for Stable Cell Imaging. ACS Appl. Bio Mater..

[75] Ansari M., Ahmad A., Kumar A., Alam P., Khan T.H., Jayamurugan G., Raza S.S., Khan R. (2021). Aminocellulose-grafted-polycaprolactone coated gelatin nanoparticles alleviate inflammation in rheumatoid arthritis: A combinational therapeutic approach. Carbohydr. Polym..

[76] Chen X., Zou J., Zhang K., Zhu J., Zhang Y., Zhu Z., Zheng H., Li F., Piao J.-G. (2021). Photothermal/matrix metalloproteinase-2 dual-responsive gelatin nanoparticles for breast cancer treatment. Acta Pharm. Sin. B.

[77] Moin A., Wani S.U.D., Osmani R.A., Abu Lila A.S., Khafagy E.-S., Arab H.H., Gangadharappa H.V., Allam A.N. (2021). Formulation, characterization, and cellular toxicity assessment of tamoxifen-loaded silk fibroin nanoparticles in breast cancer. Drug Deliv..

[78] Vallejo R., Gonzalez-Valdivieso J., Santos M., Rodriguez-Rojo S., Arias F. (2021). Production of elastin-like recombinamer-based nanoparticles for docetaxel encapsulation and use as smart drug-delivery systems using a supercritical anti-solvent process. J. Ind. Eng. Chem..

[79] Roldo M., Hornof M., Caliceti P., Bernkop-Schnürch A. (2004). Mucoadhesive thiolated chitosans as platforms for oral controlled drug delivery: Synthesis and in vitro evaluation. Eur. J. Pharm. Biopharm..

[80] Mohammed M.A., Syeda J.T.M., Wasan K.M., Wasan E.K. (2017). An Overview of Chitosan Nanoparticles and Its Application in Non-Parenteral Drug Delivery. Pharmaceutics.

[81] Sharifi-Rad J., Quispe C., Butnariu M., Rotariu L.S., Sytar O., Sestito S., Rapposelli S., Akram M., Iqbal M., Krishna A. (2021). Chitosan nanoparticles as a promising tool in nanomedicine with particular emphasis on oncological treatment. Cancer Cell Int..

[82] Yanat M., Schroën K. (2021). Preparation methods and applications of chitosan nanoparticles; with an outlook toward reinforcement of biodegradable packaging. React. Funct. Polym..

[83] Nagpal K., Singh S.K., Mishra D.N. (2010). Chitosan Nanoparticles: A Promising System in Novel Drug Delivery. Chem. Pharm. Bull..

[84] Dev A., Binulal N., Anitha A., Nair S., Furuike T., Tamura H., Jayakumar R. (2010). Preparation of poly(lactic acid)/chitosan nanoparticles for anti-HIV drug delivery applications. Carbohydr. Polym..

[85] Li P., Wang Y., Peng Z., She F., Kong L. (2011). Development of chitosan nanoparticles as drug delivery systems for 5-fluorouracil and leucovorin blends. Carbohydr. Polym..

[86] Li Y., Liu Y., Guo Q. (2021). Silk fibroin hydrogel scaffolds incorporated with chitosan nanoparticles repair articular cartilage defects by regulating TGF-β1 and BMP-2. Arthritis Res. Therapy..

[87] Zahiri M., Khanmohammadi M., Goodarzi A., Ababzadeh S., Farahani M.S., Mohandesnezhad S., Bahrami N., Nabipour I., Ai J. (2020). Encapsulation of curcumin loaded chitosan nanoparticle within poly (ε-caprolactone) and gelatin fiber mat for wound healing and layered dermal reconstitution. Int. J. Biol. Macromol..

[88] Severino P., Da Silva C.F., Andrade L.N., de Lima Oliveira D., Campos J., Souto E.B. (2019). Alginate Nanoparticles for Drug Delivery and Targeting. Curr. Pharm. Des..

[89] Almutairi F.M. (2019). Biopolymer Nanoparticles: A Review of Prospects for Application as Carrier for Therapeutics and Diagnostics. Int. J. Pharm. Res. Allied Sci..

[90] Pawar S.N., Edgar K.J. (2012). Alginate derivatization: A review of chemistry, properties and applications. Biomaterials.

[91] Oliveira D.M.L., Rezende P.S., Barbosa T.C., Andrade L.N., Bani C., Tavares D.S., da Silva C.F., Chaud M.V., Padilha F., Cano A. (2020). Double membrane based on lidocaine-coated polymyxin-alginate nanoparticles for wound healing: In vitro characterization and in vivo tissue repair. Int. J. Pharm..

[92] Li P., Dai Y.N., Wang A.-Q., Wei Q. (2008). Chitosan-Alginate Nanoparticles as a Novel Drug Delivery System for Nifedi-pine. Int. J. Biomed. Sci..

[93] Sorasitthiyanukarn F.N., Muangnoi C., Rojsitthisak P., Rojsitthisak P. (2021). Chitosan-alginate nanoparticles as effective oral carriers to improve the stability, bioavailability, and cytotoxicity of curcumin diethyl disuccinate. Carbohydr. Polym..

[94] Zohri M., Arefian E., Javar H.A., Gazori T., Aghaee-Bakhtiari S.H., Taheri M., Fatahi Y., Azadi A., Khoshayand M.R., Ghahremani M.H. (2021). Potential of chitosan/alginate nanoparticles as a non-viral vector for gene delivery: Formulation and optimization using D-optimal design. Mater. Sci. Eng. C.

[95] Zhu Z., He F., Shao H., Shao J., Li Q., Wang X., Ren H., You C., Zhang Z., Han C. (2023). Chitosan/Alginate Nanoparticles with Sustained Release of Esculentoside A for Burn Wound Healing. ACS Appl. Nano Mater..

[96] Caldonazo A., Almeida S.L., Bonetti A.F., Lazo R.E.L., Mengarda M., Murakami F.S. (2021). Pharmaceutical applications of starch nanoparticles: A scoping review. Int. J. Biol. Macromol..

[97] Campelo P.H., Sant’Ana A.S., Clerici M.T.P.S. (2020). Starch nanoparticles: Production methods, structure, and properties for food applications. Curr. Opin. Food Sci..

[98] Le Corre D., Angellier-Coussy H. (2014). Preparation and application of starch nanoparticles for nanocomposites: A review. React. Funct. Polym..

[99] Le Corre D., Bras J., Dufresne A. (2010). Starch Nanoparticles: A Review. Biomacromolecules.

[100] Alp E., Damkaci F., Güven E., Tenniswood M. (2019). Starch nanoparticles for delivery of the histone deacetylase inhibitor CG-1521 in breast cancer treatment. Int. J. Nanomed..

[101] Acevedo-Guevara L., Nieto-Suaza L., Sanchez L.T., Pinzon M.I., Villa C.C. (2018). Development of native and modified banana starch nanoparticles as vehicles for curcumin. Int. J. Biol. Macromol..

[102] Li L., He S., Yu L., Elshazly E.H., Wang H., Chen K., Zhang S., Ke L., Gong R. (2019). Codelivery of DOX and siRNA by folate-biotin-quaternized starch nanoparticles for promoting synergistic suppression of human lung cancer cells. Drug Deliv..

[103] Mariadoss A.V.A., Saravanakumar K., Sathiyaseelan A., Karthikkumar V., Wang M.-H. (2022). Smart drug delivery of p-Coumaric acid loaded aptamer conjugated starch nanoparticles for effective triple-negative breast cancer therapy. Int. J. Biol. Macromol..

[104] Nallasamy P., Ramalingam T., Nooruddin T., Shanmuganathan R., Arivalagan P., Natarajan S. (2020). Polyherbal drug loaded starch nanoparticles as promising drug delivery system: Antimicrobial, antibiofilm and neuroprotective studies. Process. Biochem..

[105] Majcher M.J., McInnis C.L., Himbert S., Alsop R.J., Kinio D., Bleuel M., Rheinstadter M., Smeets N.M., Hoare T. (2020). Photopolymerized Starchstarch Nanoparticle (SNP) network hydrogels. Carbohydr. Polym..

[106] Díaz-Montes E. (2021). Dextran: Sources, Structures, and Properties. Polysaccharides.

[107] Wasiak I., Kulikowska A., Janczewska M., Michalak M., Cymerman I.A., Nagalski A., Kallinger P., Szymanski W.W., Ciach T. (2016). Dextran Nanoparticle Synthesis and Properties. PLoS ONE.

[108] Thambi T., Gil You D., Han H.S., Deepagan V.G., Jeon S.M., Suh Y.D., Choi K.Y., Kim K., Kwon I.C., Yi G.-R. (2014). Bioreducible Carboxymethyl Dextran Nanoparticles for Tumor-Targeted Drug Delivery. Adv. Heal. Mater..

[109] Keliher E.J., Yoo J., Nahrendorf M., Lewis J.S., Marinelli B., Newton A., Pittet M.J., Weissleder R. (2011). ^89^Zr-Labeled Dextran Nanoparticles Allow in Vivo Macrophage Imaging. Bioconjugate Chem..

[110] Jamwal S., Ram B., Ranote S., Dharela R., Chauhan G.S. (2019). New glucose oxidase-immobilized stimuli-responsive dextran nanoparticles for insulin delivery. Int. J. Biol. Macromol..

[111] Butzbach K., Konhäuser M., Fach M., Bamberger D.N., Breitenbach B., Epe B., Wich P.R. (2019). Receptor-mediated Uptake of Folic Acid-functionalized Dextran Nanoparticles for Applications in Photodynamic Therapy. Polymers.

[112] Lan J., Li Y., Wen J., Chen Y., Yang J., Zhao L., Xia Y., Du H., Tao J., Li Y. (2022). Acitretin-Conjugated Dextran Nanoparticles Ameliorate Psoriasis-like Skin Disease at Low Dosages. Front. Bioeng. Biotechnol..

[113] Naha P.C., Hsu J.C., Kim J., Shah S., Bouché M., Si-Mohamed S., Rosario-Berrios D.N., Douek P., Hajfathalian M., Yasini P. (2020). Dextran-Coated Cerium Oxide Nanoparticles: A Computed Tomography Contrast Agent for Imaging the Gastrointestinal Tract and Inflammatory Bowel Disease. ACS Nano.

[114] Abid M., Naveed M., Azeem I., Faisal A., Nazar M.F., Yameen B. (2020). Colon specific enzyme responsive oligoester crosslinked dextran nanoparticles for controlled release of 5-fluorouracil. Int. J. Pharm..

[115] Arulmozhi V., Pandian K., Mirunalini S. (2013). Ellagic acid encapsulated chitosan nanoparticles for drug delivery system in human oral cancer cell line (KB). Colloids Surf. B Biointerfaces.

[116] El-Alfy E.A., El-Bisi M.K., Taha G.M., Ibrahim H.M. (2020). Preparation of biocompatible chitosan nanoparticles loaded by tetracycline, gentamycin and ciprofloxacin as novel drug delivery system for improvement the antibacterial properties of cellulose based fabrics. Int. J. Biol. Macromol..

[117] Rahbar M., Morsali A., Bozorgmehr M.R., Beyramabadi S.A. (2020). Quantum chemical studies of chitosan nanoparticles as effective drug delivery systems for 5-fluorouracil anticancer drug. J. Mol. Liq..

[118] Yu A., Shi H., Liu H., Bao Z., Dai M., Lin D., Lin D., Xu X., Li X., Wang Y. (2020). Mucoadhesive dexamethasone-glycol chitosan nanoparticles for ophthalmic drug delivery. Int. J. Pharm..

[119] Afshar M., Dini G., Vaezifar S., Mehdikhani M., Movahedi B. (2020). Preparation and characterization of sodium alginate/polyvinyl alcohol hydrogel containing drug-loaded chitosan nanoparticles as a drug delivery system. J. Drug Deliv. Sci. Technol..

[120] Zahoor A., Rajesh P., Sadhna S., Khuller G.K. (2005). Alginate Nanoparticles as Antituberculosis Drug Carriers: Formulation Development, Pharmacokinetics and Therapeutic Potential. Indian J. Chest. Dis. Allied. Sci..

[121] Sorasitthiyanukarn F.N., Muangnoi C., Rojsitthisak P., Rojsitthisak P. (2022). Chitosan oligosaccharide/alginate nanoparticles as an effective carrier for astaxanthin with improving stability, in vitro oral bioaccessibility, and bioavailability. Food Hydrocoll..

[122] Ma X., Jian R., Chang P.R., Yu J. (2008). Fabrication and Characterization of Citric Acid-Modified Starch Nanoparticles/Plasticized-Starch Composites. Biomacromolecules.

[123] Delrish E., Ghassemi F., Jabbarvand M., Lashay A., Atyabi F., Soleimani M., Dinarvand R. (2022). Biodistribution of Cy5-labeled Thiolated and Methylated Chitosan-Carboxymethyl Dextran Nanoparticles in an Animal Model of Retinoblastoma. J. Ophthalmic Vis. Res..

[124] Jafar M.M.A., Heather S., Al A.A. (2019). Functional Biopolymers.

[125] Espinoza S.M., Patil H.I., San Martin Martinez E., Casañas Pimentel R., Ige P.P. (2020). Poly-ε-caprolactone (PCL), a promising polymer for pharmaceutical and biomedical applications: Focus on nanomedicine in cancer. Int. J. Polym. Mater. Polym. Biomater..

[126] Vert M., Li S.M., Spenlehauer G., Guerin P. (1992). Bioresorbability and biocompatibility of aliphatic polyesters. J. Mater. Sci. Mater. Med..

[127] Woodruff M.A., Hutmacher D.W. (2010). The return of a forgotten polymer—Polycaprolactone in the 21st century. Prog. Polym. Sci..

[128] Sinha V.R., Bansal K., Kaushik R., Kumria R., Trehan A. (2004). Poly-ϵ-caprolactone microspheres and nanospheres: An overview. Int. J. Pharm..

[129] Łukasiewicz S., Mikołajczyk A., Błasiak E., Fic E., Dziedzicka-Wasylewska M. (2021). Polycaprolactone Nanoparticles as Promising Candidates for Nanocarriers in Novel Nanomedicines. Pharmaceutics.

[130] Alex A., Joseph A., Shavi G., Rao J.V., Udupa N. (2016). Development and evaluation of carboplatin-loaded PCL nanoparticles for intranasal delivery. Drug Deliv..

[131] Mei L., Zeng X., Chen H., Zheng Y., Song, Huang L., Ma Y. (2011). Novel docetaxel-loaded nanoparticles based on PCL-Tween 80 copolymer for cancer treatment. Int. J. Nanomed..

[132] Abriata J.P., Turatti R.C., Luiz M.T., Raspantini G.L., Tofani L.B., Amaral R.L.F.D., Swiech K., Marcato P.D., Marchetti J.M. (2019). Development, characterization and biological in vitro assays of paclitaxel-loaded PCL polymeric nanoparticles. Mater. Sci. Eng. C.

[133] El-Habashy S.E., Eltaher H.M., Gaballah A., Zaki E.I., Mehanna R.A., El-Kamel A.H. (2021). Hybrid bioactive hydroxyapatite/polycaprolactone nanoparticles for enhanced osteogenesis. Mater. Sci. Eng. C.

[134] Hao Y., Chen Y., He X., Yang F., Han R., Yang C., Li W., Qian Z. (2020). Near-infrared responsive 5-fluorouracil and indocyanine green loaded MPEG-PCL nanoparticle integrated with dissolvable microneedle for skin cancer therapy. Bioact. Mater..

[135] Shahab M.S., Rizwanullah M., Alshehri S., Imam S.S. (2020). Optimization to development of chitosan decorated polycaprolactone nanoparticles for improved ocular delivery of dorzolamide: In vitro, ex vivo and toxicity assessments. Int. J. Biol. Macromol..

[136] Chen Y., Lu Y., Hu D., Peng J., Xiao Y., Hao Y., Pan M., Yuan L., Qian Z. (2021). Cabazitaxel-loaded MPEG-PCL copolymeric nanoparticles for enhanced colorectal cancer therapy. Appl. Mater. Today.

[137] Pan Q., Tian J., Zhu H., Hong L., Mao Z., Oliveira J.M., Reis R.L., Li X. (2020). Tumor-Targeting Polycaprolactone Nanoparticles with Codelivery of Paclitaxel and IR780 for Combinational Therapy of Drug-Resistant Ovarian Cancer. ACS Biomater. Sci. Eng..

[138] Kumari A., Yadav S.K., Pakade Y.B., Kumar V., Singh B., Chaudhary A., Yadav S.C. (2011). Nanoencapsulation and characterization of Albizia chinensis isolated antioxidant quercitrin on PLA nanoparticles. Colloids Surf. B Biointerfaces.

[139] Da Costa D., Exbrayat-Héritier C., Rambaud B., Megy S., Terreux R., Verrier B., Primard C. (2021). Surface charge modulation of rifampicin-loaded PLA nanoparticles to improve antibiotic delivery in Staphylococcus aureus biofilms. J. Nanobiotechnology.

[140] Niza E., Božik M., Bravo I., Clemente-Casares P., Sánchez A.L., Juan A., Klouček P., Alonso-Moreno C. (2020). PEI-coated PLA nanoparticles to enhance the antimicrobial activity of carvacrol. Food Chem..

[141] Ghaffarzadegan R., Khoee S., Rezazadeh S. (2020). Fabrication, characterization and optimization of berberine-loaded PLA nanoparticles using coaxial electrospray for sustained drug release. DARU J. Pharm. Sci..

[142] Zhang L., Zhu H., Gu Y., Wang X., Wu P. (2019). Dual drug-loaded PLA nanoparticles bypassing drug resistance for improved leukemia therapy. J. Nanoparticle Res..

[143] Xie Z., Su Y., Kim G.B., Selvi E., Ma C., Aragon-Sanabria V., Hsieh J., Dong C., Yang J. (2017). Immune Cell-Mediated Biodegradable Theranostic Nanoparticles for Melanoma Targeting and Drug Delivery. Small.

[144] Kumar A., Han S.S. (2017). PVA-based hydrogels for tissue engineering: A review. Int. J. Polym. Mater. Polym. Biomater..

[145] Kumar A., Negi Y.S., Choudhary V., Bhardwaj N.K. (2014). Microstructural and mechanical properties of porous biocomposite scaffolds based on polyvinyl alcohol, nano-hydroxyapatite and cellulose nanocrystals. Cellulose.

[146] Rivera-Hernández G., Antunes-Ricardo M., Martínez-Morales P., Sánchez M.L. (2021). Polyvinyl alcohol based-drug delivery systems for cancer treatment. Int. J. Pharm..

[147] El-Dafrawy S.M., Tarek M., Samra S., Hassan S.M. (2021). Synthesis, photocatalytic and antidiabetic properties of ZnO/PVA nanoparticles. Sci. Rep..

[148] Li J.K., Wang N., Wu X.S. (1998). Poly(vinyl alcohol) nanoparticles prepared by freezing–thawing process for protein/peptide drug delivery. J. Control. Release.

[149] Byun Y., Hwang J.B., Bang S.H., Darby D., Cooksey K., Dawson P.L., Park H.J., Whiteside S. (2011). Formulation and characterization of α-tocopherol loaded poly ε-caprolactone (PCL) nanoparticles. LWT-Food Sci. Technol..

[150] Joye I.J., McClements D.J. (2014). Biopolymer-based nanoparticles and microparticles: Fabrication, characterization, and application. Curr. Opin. Colloid Interface Sci..

[151] Kakran M., Antipina M.N. (2014). Emulsion-based techniques for encapsulation in biomedicine, food and personal care. Curr. Opin. Pharmacol..

[152] Jin H.Y., Xia F., Zhao Y.P. (2012). Preparation of hydroxypropyl methyl cellulose phthalate nanoparticles with mixed solvent using supercritical antisolvent process and its application in co-precipitation of insulin. Adv. Powder Technol..

[153] Jones O.G., McClements D.J. (2010). Biopolymer Nanoparticles from Heat-Treated Electrostatic Protein-Polysaccharide Complexes: Factors Affecting Particle Characteristics. J. Food Sci..

[154] Vecchione D., Grimaldi A.M., Forte E., Bevilacqua P., Netti P.A., Torino E. (2017). Hybrid Core-Shell (HyCoS) Nanoparticles produced by Complex Coacervation for Multimodal Applications. Sci. Rep..

[155] Schmitt C., Turgeon S.L. (2011). Protein/polysaccharide complexes and coacervates in food systems. Adv. Colloid Interface Sci..

[156] Bock N., Woodruff M.A., Hutmacher D.W., Dargaville T.R. (2011). Electrospraying, a Reproducible Method for Production of Polymeric Microspheres for Biomedical Applications. Polymers.

[157] Ma J., Lee S.M.-Y., Yi C., Li C.-W. (2017). Controllable synthesis of functional nanoparticles by microfluidic platforms for biomedical applications—A review. Lab Chip.

[158] Xu S., Nie Z., Seo M., Lewis P., Kumacheva E., Stone H.A., Garstecki P., Weibel D.B., Gitlin I., Whitesides G.M. (2005). Generation of Monodisperse Particles by Using Microfluidics: Control over Size, Shape, and Composition. Angew. Chem. Int. Ed..

[159] Krishnadasan S., Brown R.J.C., Demello A.J., Demello J.C. (2007). Intelligent routes to the controlled synthesis of nanoparticles. Lab Chip.

[160] Gómez-Guillén M.C., Montero M.P. (2021). Enhancement of oral bioavailability of natural compounds and probiotics by mucoadhesive tailored biopolymer-based nanoparticles: A review. Food Hydrocoll..

[161] Lynch C.R., Kondiah P.P.D., Choonara Y.E. (2021). Advanced Strategies for Tissue Engineering in Regenerative Medicine: A Biofabrication and Biopolymer Perspective. Molecules.

[162] Banerjee A., Bandopadhyay R. (2016). Use of dextran nanoparticle: A paradigm shift in bacterial exopolysaccharide based biomedical applications. Int. J. Biol. Macromol..

